# CRISPR-Cas9 enrichment and long read sequencing for fine mapping in plants

**DOI:** 10.1186/s13007-020-00661-x

**Published:** 2020-09-01

**Authors:** Elena López-Girona, Marcus W. Davy, Nick W. Albert, Elena Hilario, Maia E. M. Smart, Chris Kirk, Susan J. Thomson, David Chagné

**Affiliations:** 1 The New Zealand Institute for Plant and Food Research Limited (Plant & Food Research), Private Bag 11600, Palmerston North, 4442 New Zealand; 2 Plant & Food Research, Te Puke, 3182 New Zealand; 3 Plant & Food Research, Auckland, 1142 New Zealand; 4 Plant & Food Research, Lincoln, 8140 New Zealand

**Keywords:** Causative variant, SNP, Apple, *MYB10*, Red flesh, Oxford nanopore, QTL cloning

## Abstract

**Background:**

Genomic methods for identifying causative variants for trait loci applicable to a wide range of germplasm are required for plant biologists and breeders to understand the genetic control of trait variation.

**Results:**

We implemented Cas9-targeted sequencing for fine-mapping in apple, a method combining CRISPR-Cas9 targeted cleavage of a region of interest, followed by enrichment and long-read sequencing using the Oxford Nanopore Technology (ONT). We demonstrated the capability of this methodology to specifically cleave and enrich a plant genomic locus spanning 8 kb. The repeated mini-satellite motif located upstream of the *Malus* × *domestica* (apple) *MYB10* transcription factor gene, causing red fruit colouration when present in a heterozygous state, was our exemplar to demonstrate the efficiency of this method: it contains a genomic region with a long structural variant normally ignored by short-read sequencing technologies

Cleavage specificity of the guide RNAs was demonstrated using polymerase chain reaction products, before using them to specify cleavage of high molecular weight apple DNA. An enriched library was subsequently prepared and sequenced using an ONT MinION flow cell (R.9.4.1). Of the 7,056 ONT reads base-called using both Albacore2 (v2.3.4) and Guppy (v3.2.4), with a median length of 9.78 and 9.89 kb, respectively, 85.35 and 91.38%, aligned to the reference apple genome. Of the aligned reads, 2.98 and 3.04% were on-target with read depths of 180 × and 196 × for Albacore2 and Guppy, respectively, and only five genomic loci were off-target with read depth greater than 25 × , which demonstrated the efficiency of the enrichment method and specificity of the CRISPR-Cas9 cleavage.

**Conclusions:**

We demonstrated that this method can isolate and resolve single-nucleotide and structural variants at the haplotype level in plant genomic regions. The combination of CRISPR-Cas9 target enrichment and ONT sequencing provides a more efficient technology for fine-mapping loci than genome-walking approaches**.**

## Background

Representatives of most plant species important to primary industries have been fully sequenced following the dramatic reduction of sequencing cost. However, the individuals from which reference genomes have been assembled are often chosen because they are highly homozygous (doubled haploids or inbred lines). Typically, they do not represent the full complement of genes and variability of the germplasm that is used for selective breeding. Indeed, plant breeders often use wild, undomesticated relatives as donors of alleles for pest and disease resistance, as well as novel quality traits. The genomes of these wild relatives are often even further substantially different, in both sequence and structure, from the reference species’ genomes. To address this issue, researchers have focused on cataloguing the most abundant genomic variations present on any genome—single nucleotide polymorphisms (SNPs)—using the reference genomes as guides for aligning (re)sequencing data. SNPs can be readily identified and visualized using SNP arrays or short-read sequencing approaches, such as Whole Genome Sequencing (WGS), or restriction-enzyme-based enrichment approaches, such as Genotyping by Sequencing (GBS) [1] or restriction site associated DNA (RAD) tags [2]. However, a growing body of evidence demonstrates that there are other types of genomic variants [e.g. structural variants (SVs), copy number variations (CNV) and epimutations] that are also directly causative for key traits. These genomic variants can have complex allelic diversity, which is not reliably captured by the typical read length (several hundred bases) of short-read sequencing technologies. Sequence assembly of these areas is hampered by repeats and transposable elements, which is an issue when using short-read sequencing methods.

Not only do the causative variants for key plant breeding trait loci provide ideal markers for efficient selection, but developing an understanding of the nature of the genetic variants may allow similar alleles to be generated by gene-editing methods in species other than the original host, enabling the introduction of valuable new traits into these crops. However, identification of such causative variants has been hampered due to limitations of using SNPs, which do not encode allelic variation in all traits. New methods to identify variants that are applicable to a wide range of germplasm are required. While WGS and alignment to a reference genome approach is possible, this often relies on massive paralleled short-read sequencing, which suffers from low sensitivity (30–70%) and a high false discovery rate (up to 85%) of SVs [3] because they are inferred directly from aberrant short-read alignments based on the identification of an unexpected depth of coverage, or inconsistent orientation or distance between the alignment of paired-end reads [4, 5]. Targeted capture by in-solution hybridization of coding and non-coding regions can be more cost-effective for the identification of variants, especially for very large, repetitive genomes [6, 7]. Capture-based methods, however, can only infer SVs by analysing the depth of coverage of those sequenced regions [8]. Complete de novo haplotype-resolved assembly is considered the best approach for the full characterization of the genetic diversity, however, this method struggles to identify low-copy repeats, also known as segmental duplications [9]. Targeted approaches employing long-read sequencing (LRS) are therefore an attractive alternative. The main advantage of LRS compared with short-read sequencing is that long reads (> 10 kb on average) can be obtained from single DNA molecules in real time without polymerase chain reaction (PCR) amplification. Although long-fragment targeted capture methods have been developed in plants, they are based on in-solution hybridization of predesigned long probes from a reference genome and the enrichment of the targeted regions via post-capture PCR amplification before sequencing [10–13], which generates biases from both the reference genome and the use of PCR. The sequencing of native molecules (both DNA and RNA) based on Oxford Nanopore Technology (ONT) and the preparation of a sequencing library without PCR amplification would be preferable. Such an approach is possible using the CRISPR-Cas9 system.

Clustered regularly interspaced short palindromic repeat (CRISPR) is a bacterial and archaeal defence system against foreign nucleic acids from invading viruses and plasmids, via nucleases, such as Cas9 that target specific sequences [14–16]. The most studied CRISPR system is the type II CRISPR-Cas system from *Streptococcus pyogenes*, which relies on only a single protein, the nuclease Cas9, and two non-coding RNAs, a 20-bp crRNA and a tracrRNA to target DNA. These two RNAs are fused into an artificial short guide RNA (sgRNA). The combination of the Cas9 protein and the sgRNA produces a Cas9/sgRNA complex, which binds on double-stranded DNA by the match-recognition of the first 17–20 nucleotides of the sgRNA if the target sequence presents a protospacer adjacent motif (PAM). Once bound, both Cas9 cleaves each of the DNA strands independently at three bases upstream of the PAM, generating a blunt and double DNA strand end [17, 18]. In addition to being widely adopted as a tool for genome editing [18–21] and for epigenome remodelling, when dead Cas9 (dCas9) is used [22], CRISPR/Cas9 is also a valuable tool for in vitro DNA cleavage, where it can act as a precise, custom restriction endonuclease, enabling CRISPR methods for targeted sequencing [23].

The use of CRISPR/Cas9 for in vitro DNA cleavage purposes was first reported in 2015 for Cas9-assisted targeting of chromosome segments (CATCH) for cloning of large intact bacterial genomic DNA fragments (up to 100 kb) via Gibson assembly [24], and for transformation-associated recombination (TAR)-cloning in yeast of large human genomic sequences (up to 150 kb) [25]. Optimization of the CATCH protocol was subsequently achieved by performing in-gel cleavage and the separation of the target region from the rest of the genomic DNA by pulse field gel electrophoresis [26, 27]. However, it was the utility of CATCH coupled with targeted ONT sequencing that turned the method into a powerful tool for the isolation, in real time, of very large native nucleotide molecules. This method was first developed to isolate a human breast cancer gene (*BRCA1*), obtaining sufficient coverage (70 × on average) to characterize variants, albeit only 1% of the reads were on target [28]. Another approach includes the use of dCas9 to ‘pull down’ targeted regions by using streptavidin magnetic beads to attach to the tracrRNA and ligate to ONT adapters [29]. An updated method for effective targeted enrichment was proposed [30], which started with the dephosphorisation of the genomic DNA, followed by targeted CRISPR-Cas9 cleavage, which enabled the specific ligation of ONT adapters to the digested ends. A similar method has been reported for human diagnostic purposes, targeting genes in order to identify variants associated with a range of diseases [31, 32].

**Fig. 1:**
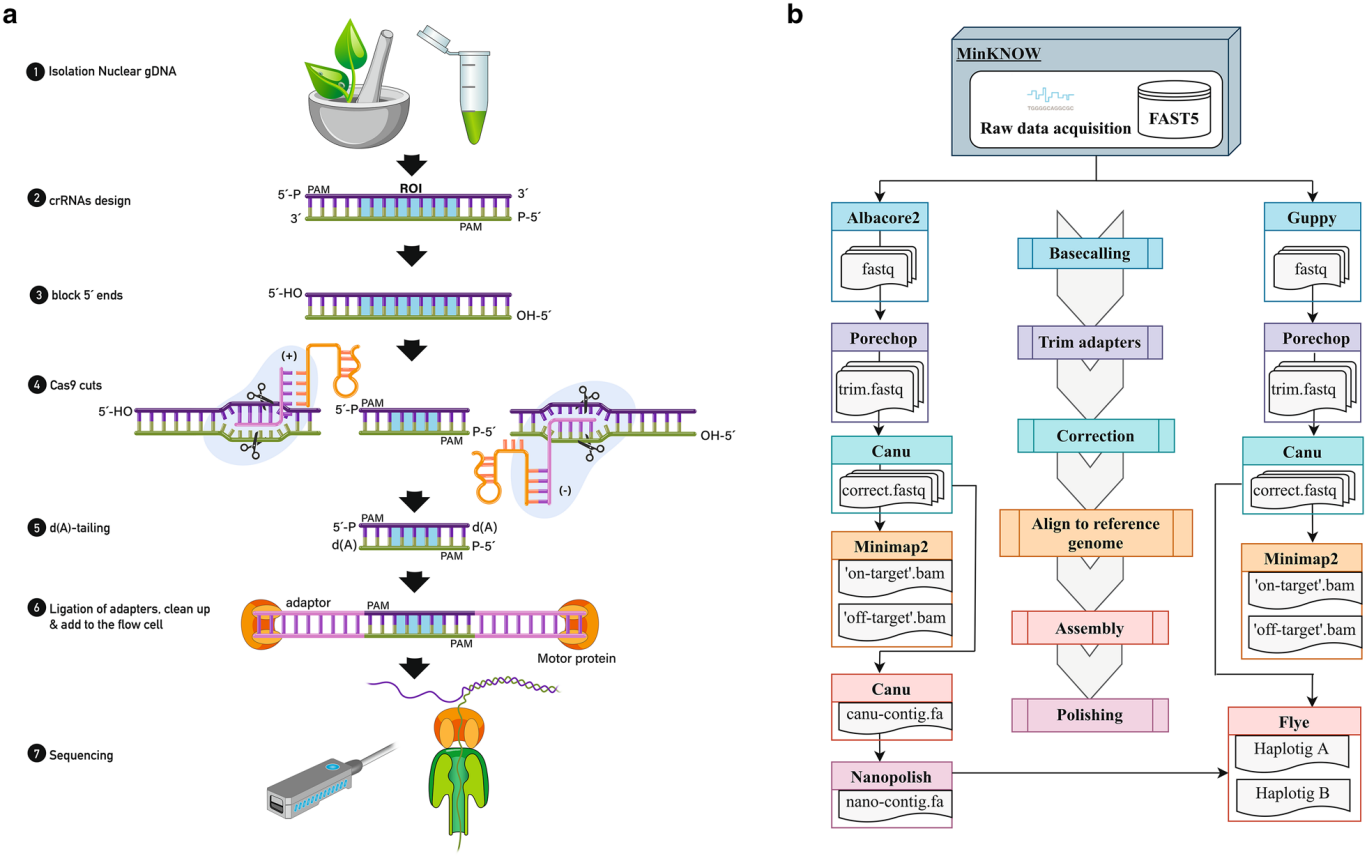
**a** In vitro CRISPR-Cas9 enrichment steps. High molecular weight nuclear DNA is extracted and crRNA probes designed. DNA 5′ ends are dephosphorylated to reduce ligation of sequencing adapters to non-target strands. Cas9 ribonucleoprotein particles (RNPs) bind at each side and cleave the region of interest (ROI). Double strand DNA is cleaved by Cas9 revealing blunt ends with ligatable 5′ phosphates. Cas9 remains bound to the protospacer adjacent motif (PAM)-distal end giving directionality for the strands towards the ROI. All DNA is dA-tailed, preparing the blunt ends for sequencing adapter ligation. ONT adapters are ligated to Cas9 cut sites which are both 3′ dA-tailed and 5′ phosphorylated. Library is cleaned to remove excess adapters using AMPure XP beads. Non-target molecules are not removed from the library. Library is added to the flow cell for sequencing. **b** Bioinformatics pipeline. Raw FAST5 reads are base called using Albacore2 (v2.3.4, ONT) and Guppy (v3.2.4, ONT) and converted to FASTQ. Reads get adapters trimmed using Porechop (v0.2.3 https://github.com/rrwick/Porechop), corrected using Canu (v1.7) [36] and aligned to the apple reference genome (‘Golden Delicious’ double haploid GDDH13v1.1) [34] using Minimap2 (v2.9) [46] to localize physically the ‘on-target’ and ‘off-target’ enriched regions. A de novo assembly is performed by Canu (v1.7) [36] using Albacore2 (v2.3.4, ONT) corrected reads and polished using Nanopolish (v0.11.1) [37]. Canu_corrected reads and the Canu_nanopolished assembly are used as inputs to run the final assembly performed by Flye (v2.5) [38]

Here, we used CRISPR-Cas9 targeted cleavage for the isolation of a region of interest, followed by enrichment and LRS using ONT (Fig. 1). This method does not require sequencing the entire genome and uses techniques and hardware commonly available in a molecular biology laboratory. We validated this method for use in fine-mapping by isolating the causative variant for red fruit coloration in apple (Type 1 red flesh), previously discovered to be a repeated mini-satellite motif located upstream of the *MYB10* transcription factor open reading frame [33].

## Results

### Assessment of guide RNA activity and specificity using an amplicon cleavage test

Four guide RNAs (gRNAs) were designed to test whether CRISPR/Cas9 could specifically excise a 7841 base pair (bp) segment of the Type 1 apple red flesh *MYB10* gene locus. Of these, two gRNAs specifically targeted the upstream promotor region and two targeted regions downstream of the gene. The four gRNAs had 100% specificity scores (Table 1) for the production of specific fragment lengths based on the apple reference genome assembly (*Malus* × *domestica* ‘Golden Delicious’ double haploid GDDH13v1.1) [34]. Sanger sequencing of PCR products containing the gRNA targets amplified from DNA of the Type 1 red flesh apple accession ABGS0131 was used to assess whether variants of the gRNA target sequences were present.

**Table 1:** CRISPR RNA oligonucleotide sequences used for cleavage of genomic DNA in the apple *MYB10* locus

Name	Sequence 5′-3′	Off-target score (%)	On-target activity score (%)	Chr	Start position	End position
crRNA_RF_1_F	GTCATATCTAAGGACCCGCGTGG	100	76.30	Chr09	35542701	35542723
crRNA_RF_2_F	TCTGTACTCCGTCTGTCGGTCGG	100	77.90	Chr09	35542848	35542870
crRNA_RF_3_R	AGAAGACTGTCAATCCCGAGTGG	100	79.60	Chr09	35550689	35550711
crRNA_RF_4_F	TGTCTGGAAAGTTTCTAACGCGG	100	70.80	Chr09	35551878	35551900

Off-target score (specificity) for guide RNA sequences were calculated according to [51] against (‘Golden Delicious’ double haploid GDDH13v1.1) [34], with a score of 100% predicting low off target activity. On target activity scores (efficiency) were calculated according to [52].

**Fig. 2:**
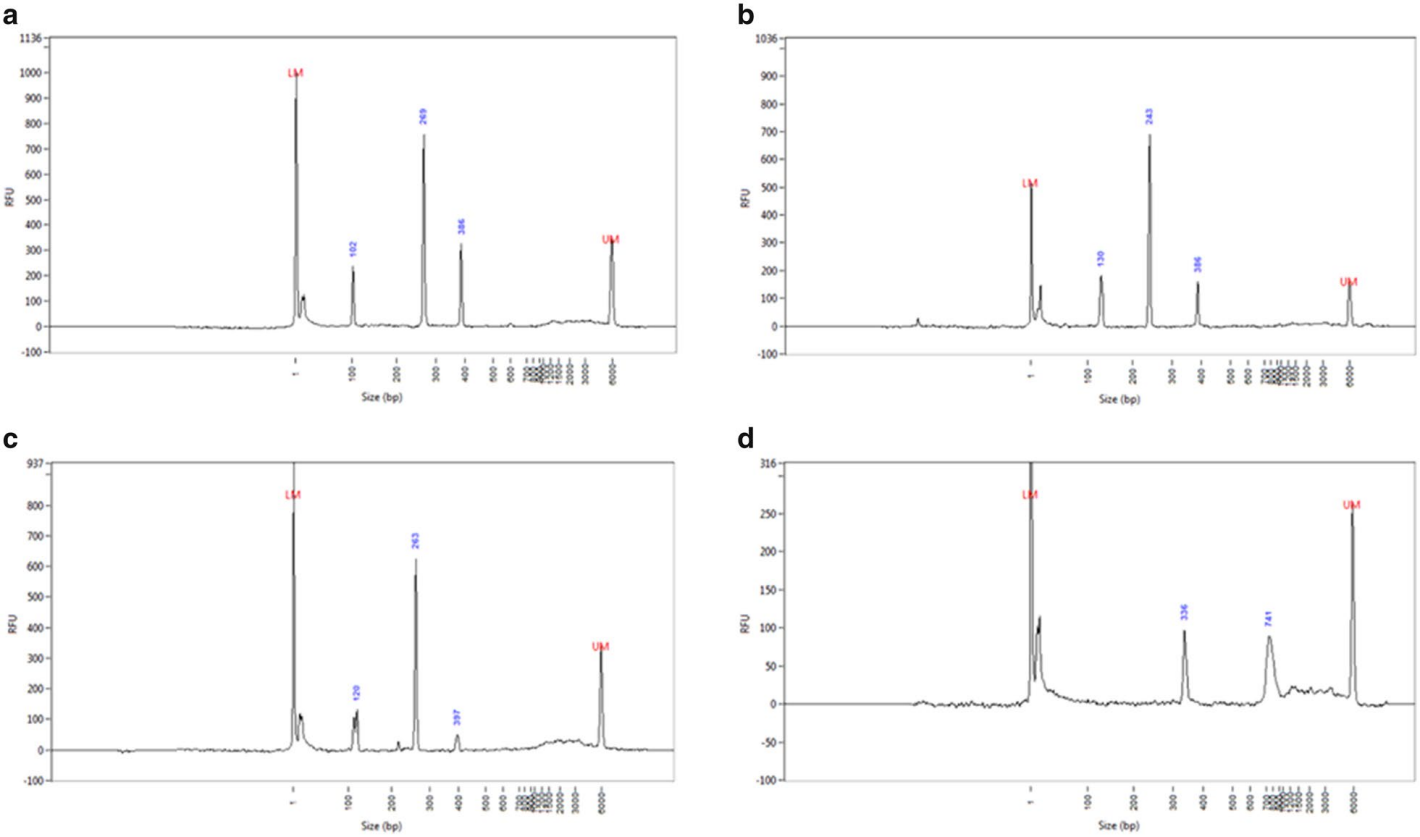
Guide RNA Cas9 digestion efficiency in PCR-amplicons linked to the apple *MYB10* locus. Fragment analysis evaluating the four gRNAs designed: graphics A to D are for crRNA_RF_1 to 4, respectively. The first two peaks on A–C correspond to both fragments after Cas9 digestion. D shows a single digested fragment because the digestion occurred in the middle of the original PCR amplicon. The longest fragments correspond to undigested original PCR amplicons. Lower marker (LM) set to 1 bp and 6000 bp upper marker (UM) from the high sensitivity NGS fragment analysis kit (1–6000 bp), DNF-474, Advance Analytical

Cleavage specificity was tested using PCR products (Table 2) from ABGS0131. The cleavage products of the four different gRNAs (Table 1) visualized on a Fragment Analyser (Advanced Analytical Technologies, Inc.) showed the expected fragment sizes after digestion of each amplicon (Fig. 2). There was a SNP present between two of the four gRNAs and the ABGS0131 sequence (the bolded “G” in crRNA_RF_2F: “TCTGTACTCCGTCTGTCGGTC**G**G” and “T” in crRNA_RF_4F: “TG**T**CTGGAAAGTTTCTAACGCGG” replaced by “A” in the ABGS0131 sequence). However, since successful digestion of all amplicons was observed regardless of the SNPs, both crRNAs were used for subsequent experiments.

**Table 2:** Primer sequences used for PCR amplification of CRISPR/Cas9 target sites of the apple *MYB10* locus

Name	crRNA	Sequence 5′-3′	Tm	%GC	Start position	End position
Chr9_35542587_F	crRNA_RF_1, crRNA_RF_2	AACAAGATGATGACGACGTG	56.2	45	35542587	35542606
Chr9_35542966_R	crRNA_RF_1, crRNA_RF_2	GATGCACGAACTGATACTGT	55.6	45	35542947	35542966
Chr9_35550584_F	crRNA_RF_3	CCCTGTATGCGAAAGACAAT	55.8	45	35550584	35550603
Chr9_35550962_R	crRNA_RF_3	AAAAGACCACATGCATGCTG	57.3	45	35550943	35550962
Chr9_35551563_F	crRNA_RF_4	TGATTGAATGTCTCCACCA	53.6	42.1	35551563	35551581
Chr9_35552158_R	crRNA_RF_4	CACATGTGAGAGAGATTTGC	54.4	45	35552158	35552177

*crRNA* CRISPR RNA, *Tm* melting temperature (°C), *%GC* GC content, start and end positions are in base pairs on apple chromosome 9 (‘Golden Delicious’ double haploid GDDH13v1.1) [34].

### Long-read sequencing of enriched libraries

**Fig. 3:**
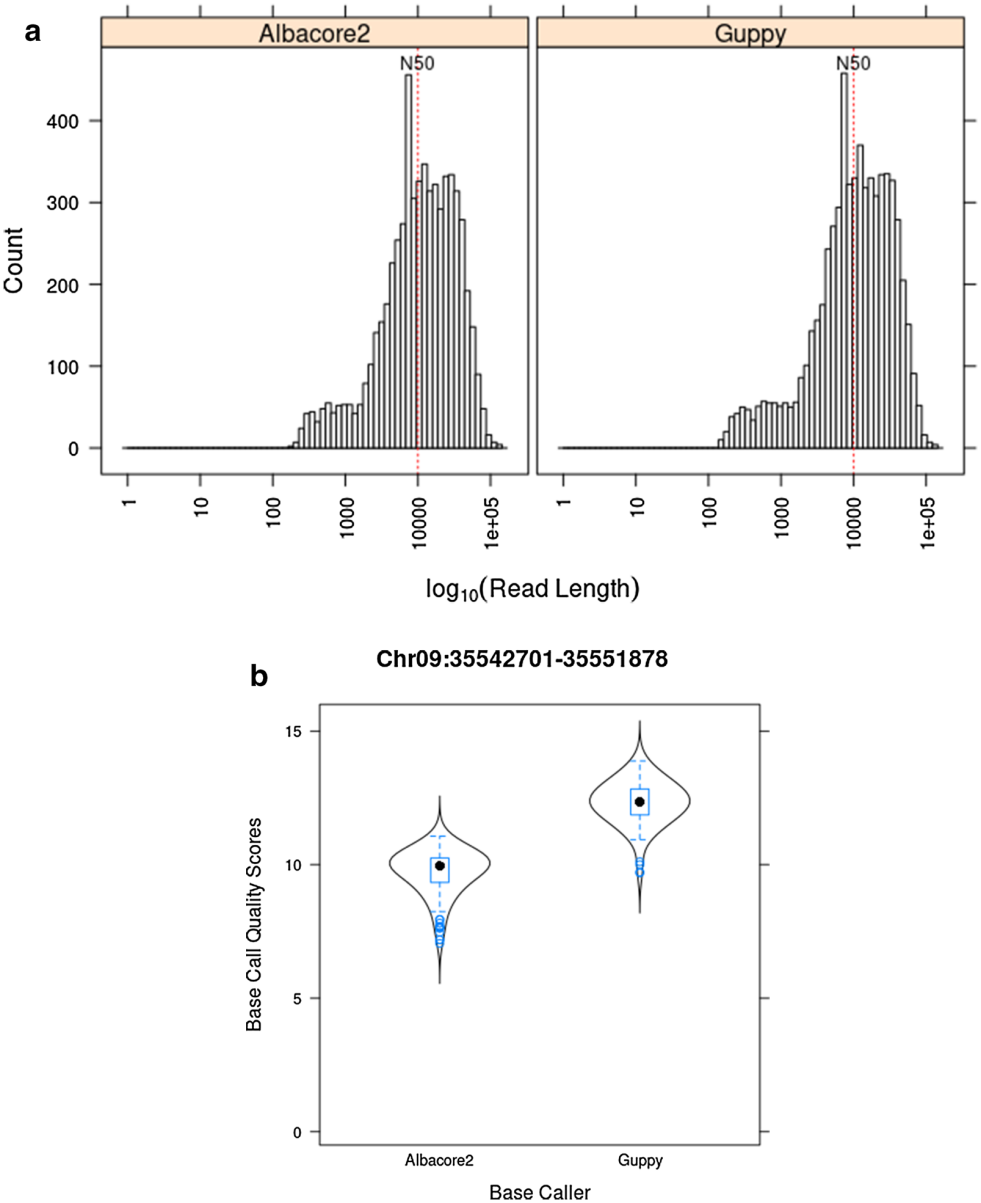
Read length and quality statistics for Oxford Nanopore Technology sequencing. **a** Read length statistics: log-transformed histogram of read length from base-called reads coming from Albacore (v2.3.4, ONT) and Guppy (v3.2.4, ONT) base callers. Vertical blue line indicates the read N50. **b** Average quality score distribution of ‘on-target’ reads coming from Albacore2 and Guppy base callers

Enriched libraries from ABGS0131 genomic DNA were prepared and sequenced using the ONT Ligation Sequencing Kit (catalog no. SQL-LSK109) and a single ONT MinION flow cell (R.9.4.1). A total of 7056 reads were base-called using Albacore (v2.3.4, ONT) and Guppy (v3.2.4, ONT). The N50 values were similar for both base callers: 30,910 and 30,825 bp for Albacore2 and Guppy, respectively (Table 3). The read length distribution histogram exhibited a high proportion of sequenced reads close to 8 kb in length, corresponding with the targeted locus length of 7841 bp (Fig. 3a). The average read quality distribution ranged from 7.05 to 11.07 and from 9.69 to 13.88, and the median read quality was 9.95 and 12.36 for Albacore2 and Guppy, respectively (Fig. 3b).

**Table 3:** Oxford Nanopore Technology sequencing reads base-called using Albacore2 and Guppy

	Base-called reads		Bases		Median read length		N50		Median read quality	
	All	Pass	All	Pass	All	Pass	All	Pass	All	Pass
Albacore2	7056	6082	112578716	106589486	9783.5	11359	30416	30910	9.11	9.28
Guppy	7056	6377	113972292	109479250	9891.5	11008	30635	30825	11.22	11.39

Pass reads are those with a mean quality of 7 or more. Software versions: Albacore (v2.3.4, ONT) and Guppy (v3.2.4, ONT).

**Fig. 4:**
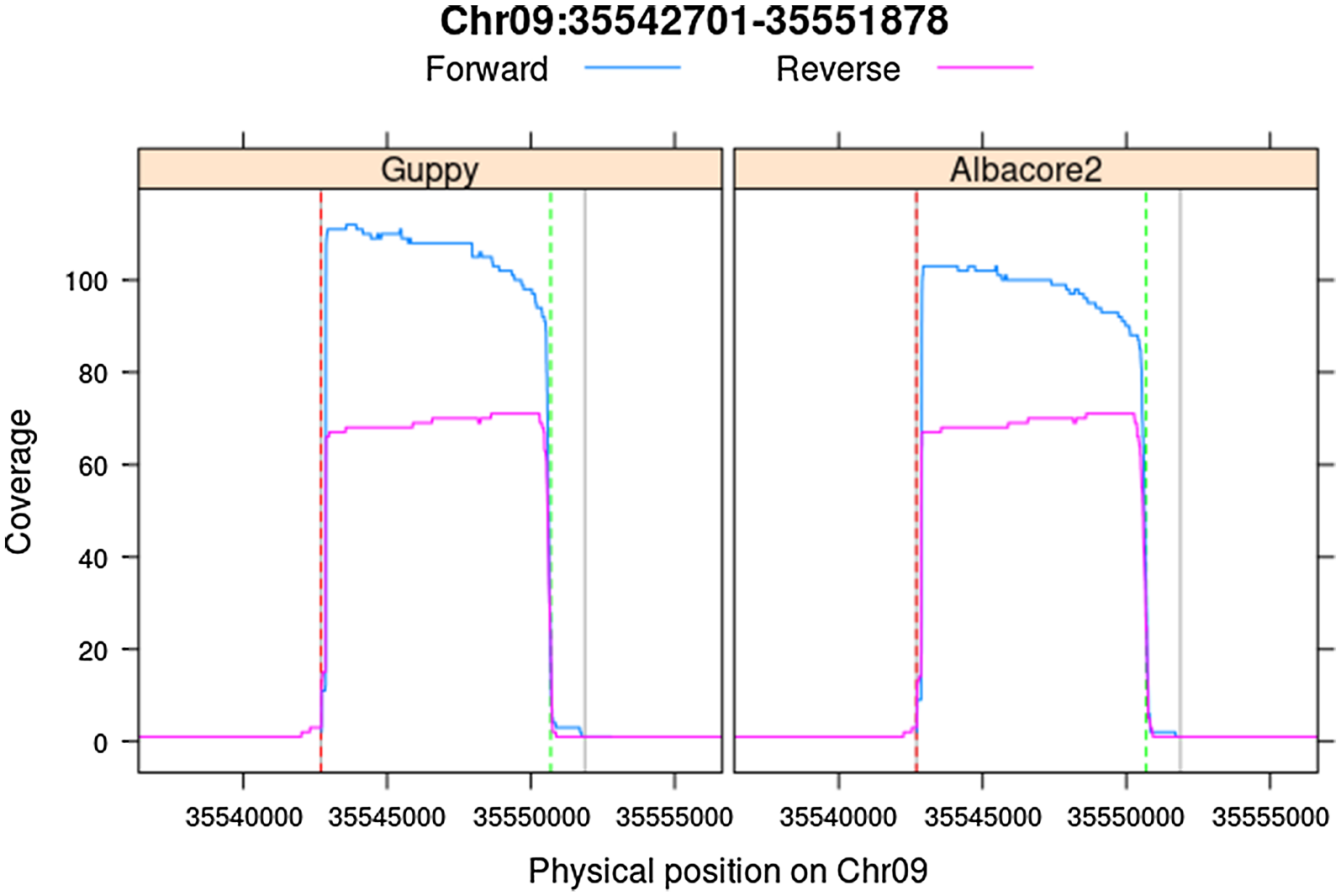
Sequencing coverage plots at the apple *MYB10* locus. Physical locations of crRNA_RF_2_F and crRNA_RF_3_R in the targeted locus on apple chromosome 9 (‘Golden Delicious’ double haploid GDDH13v1.1) [34] are shown by the dotted vertical red and green lines respectively. Physical locations of crRNA_RF_1_F and crRNA_RF_4_F are shown by grey vertical lines

The ONT reads were mapped to the ‘Golden Delicious’ double haploid GDDH13v1.1 apple reference genome [34]. A total of 6022 (85.35%) and 6448 (91.38%) reads were successfully aligned with average mapping qualities of 12.9 and 20.7 using Albacore2 and Guppy, respectively. The percentage of ‘on-target’ reads was 2.98 and 3.04% and the coverage was 180 × and 196 × for Albacore2 and Guppy, respectively (Table 4; Fig. 4). Two of the crRNAs were present in these sequences (crRNA_RF_1F and crRNA_RF_3R), however, the two crRNAs with SNPs (crRNA_RF_2F and crRNA_RF_4F) were not retrieved. In both alignments, reads containing either the R1 (a repeat unit, present in both white- and red-fleshed varieties) or R6 (six repeat units, present in heterozygous state in red-fleshed varieties) promoter alleles of the *MYB10* [33] gene could be observed when loaded onto the integrative genome viewer (IGV) [35] without performing any structural variant calling.

**Table 4:** Enrichment statistics of Oxford Nanopore Technology sequencing reads against the target apple *MYB10* locus

	Aligned reads	On-target reads		On-target coverage	On-target percentage
		Forward	Reverse		
Albacore2	6022	107	73	180	2.98%
Guppy	6448	121	75	196	3.04%

Total aligned read count against the apple genome (GDDH13v1.1), on-target reads (within 9177 bp on targeted locus), on-target total coverage and percentage.

Only four genomic regions had ‘off-target’ sequences with a read depth greater than 25 × . These regions, located on chromosomes 0, 14, 15 and 16, had small fragment lengths ranging between 795 and 8496 bp (Table 5). The largest ‘off-target’ region on chromosome 16 was only detected using Guppy base-called reads and spanned two predicted gene models of unknown functions. None of the four crRNA sequences were present in these ‘off-target’ regions (Additional file 1: Table S1 and Additional file 2: Figure S1).

**Table 5:** Off-target enrichment statistics of Oxford Nanopore Technology sequencing reads against the apple genome

Base-caller	Chromosome	Start (bp)	End (bp)	Total size (bp)	Maximum coverage	Annotation
Guppy	Chr00	48263504	48264621	1117	28	within MD00G1202200
Guppy	Chr14^a^	30289152	30291917	2765	26	2,351 bp upstream of MD14G1221200
Albacore2	Chr14^a^	30289890	30291921	2031	26	
Guppy	Chr15	47766093	47766888	795	26	within MD15G1386400
Guppy	Chr16	38910980	38919476	8496	41	spanning two genes: MD16G1282900 & MD16G1283000

Off-targets sites presented had mapping quality equal or higher than 40 × and coverage equal or higher than 25 × .

*bp*: base pairs

^a^Both chromosome 14 regions are the same. The apple reference genome (‘Golden Delicious’ double haploid GDDH13v1.1) [34] was used as reference.

### De novo assembly of the Type 1 apple red flesh locus *MYB10*

A de novo assembly was built using the Albacore2-called reads. The 6105 reads were corrected and assembled using Canu (v1.7) [36] and further improved using Nanopolish (v0.11.1) [37]. The assembly was composed of a total of seven contigs with lengths before polishing of 130,229, 17,146, 14,585, 16,132, 8006, 7927 and, 7197 bp.

Alignment against the apple reference genome showed that five of these contigs had a mapping quality < 6. Two of them had mapped reads with quality ≥ 60. One of these was a contig of 130,229 bp, which mapped partially (120 kb) to chromosome 3, however, this mapping contained many gaps and the maximum coverage was lower than 15 × . Only the 8006 bp contig (hereafter called “canu-contig”) aligned to the target *MYB10* locus. The canu-contig contained the complete R6 motif upstream of the *MYB10* locus and was flanked by the gRNA target sequences (crRNA_RF_1F and crRNA_RF_3R). After polishing, the canu-contig lost 95 bp in total, which corresponded to the repeat units 3b, 5, 4 and 3a of the R6 motif (Additional file 3: Figure S2). Coverage across the canu-contig had an average and maximum coverage of 128 × and 132 × , respectively (Additional file 4: Figure S3), and only the first 155 bp had a coverage less than 35 × . No other contig mapped to the *MYB10* target region, which indicated the 8006 bp of canu-contig was a consensus sequence that had both haplotypes collapsed. Both haplotypes were successfully split by Flye (v2.5) [38] using Canu-corrected reads and the Canu_nanopolished assembly together as input. The two split haplotigs corresponded to both haplotypes of the *MYB10* locus in their reverse and complement orientation. The haplotigs obtained were 7915 and 8029 bp long, and contained the full R1 and R6 motif of the *MYB10* locus, respectively (Additional file 5: Figure S4) and where identical to the alleles cloned by Espley et al*.* [33].

## Discussion

We have demonstrated the utility of the CRISPR-Cas9 targeted enrichment method followed by Oxford Nanopore Technology LRS for targeted genome sequencing and allele mining in a plant species. Proof-of-concept was demonstrated by retrieving the causative variant for the Type 1 red flesh locus in apple [39], the R6 motif that is located in the promoter region of the *MYB10* transcription factor gene and derived from *M.* × *domestica ‘Niedzwetzkyana’*. Enrichment was achieved by cleaving previously dephosphorylated nuclear DNA using two Cas9/guide RNA ribonucleoprotein complexes flanking the *MYB10* locus and spanning the R6 motif [33]. The cleavage of Cas9 leaves a 5′-phosphate group on DNA ends ensuring that the ligation of ONT sequencing adapters is preferential to the region of interest.

Native DNA strands enriched for the *MYB10* locus were sequenced in a single MinION ONT sequencing run without the necessity of performing PCR amplification, allowing the possibility of detecting any modified nucleotides from the raw electrical data. The efficiency of this enrichment protocol was demonstrated by the high percentage of reads mapping to the target region (~ 3% for both base calling software packages) and the high read depth in that region (greater than 180 × for both base calling software packages). The enriched ‘on-target’ region had much higher read depth than off-target regions. The ‘on-target’ region was enriched by just one of the two pairs of crRNAs used in this experiment and further confirmed by examining the sequence content of the contigs constructed by de novo assembly. Regarding the other pair of crRNAs, the low cleavage of crRNA_RF_2_F, was caused by the presence of a SNP that disrupted the target PAM, where the bolded “G” in crRNA_RF_2F: “TCTGTACTCCGTCTGTCGGTC**G**G” was replaced by “A” in the ABGS0131 sequence. In the case of crRNA_RF_4_F, two factors might have affected its cleavage efficiency. Firstly, the presence of a SNP in the protospacer sequence, which, although being a less severe modification than those occurring at the PAM, may still remove the target locus for ‘off-target’ cleavage. Secondly, the forward orientation of this crRNA that provides directionality for the strands away from the region of interest as Cas9, remains tightly bound after cleavage at the PAM-distal end, blocking the ligation of ONT adapters. With the SNP presence in the protospacer sequence, some strands might have been released from the Cas9′s bond with the result that they were ligated with adapters. ‘Off-target’ reads were distributed randomly across the genome for both base callers (data not shown). None of the four crRNA sequences were found in any ‘off-target’ regions that had a read depth greater than 25 × , indicating that they arose during ligation of ONT adapters to random exposable ends that were not dephosphorylated during the library preparation. One region had a read depth of 41 × and spanned a large fragment (> 8 kb), however this region was not detected when using the Albacore2 (v2.3.4, ONT) base caller software. Remarkably, all sequence reads in the enriched region aligned to the correct chromosome 9 region upstream of the *MYB10* gene, however, the reads did not align with the homologous duplicated region on chromosome 17 [40], upstream of *MYB110* [41], demonstrating the high fidelity of the technique.

ONT sequencing has been documented as being error-prone, requiring careful error correction, usually through use of high sequencing depth or polishing using more accurate short reads. The read depth obtained by our enrichment method was sufficient to error correct the raw reads by computing k-mer counts in preparation to computing overlaps and producing consensus sequences as performed by the Canu (v1.7) [36] correction step. The first de novo assembly attempt that was performed under standard parameters (see methods) collapsed the two haplotypes of the target region into a single contig. This was expected as most assembly software aims to produce haploid genome assemblies, thus ignoring or collapsing allelic variants together that may be involved in important biological functions. Hence, haplotype phasing becomes a required post-processing assembly step when dealing with diploid or polypoid genomes: a computationally intensive task if allowing for several degrees of divergence within the algorithms in order to maintain haplotype separation and allow for the correct error rate.

Although Cas9-enriched data require much fewer computing resources than that for analysing whole genome sequencing data, we found that Canu took several days to build the first assembly. Therefore, we also used Flye assembler (v2.5) [38], which was able to generate two haplotigs that corresponded with the two haplotypes found at the *MYB10* locus on the sequenced Type 1 red flesh accession ABGS0131 in a much shorter run time and without the necessity of enabling the option to retain alternative haplotypes (–keep-haplotypes) included in the latest version of this software.

**Fig. 5:**
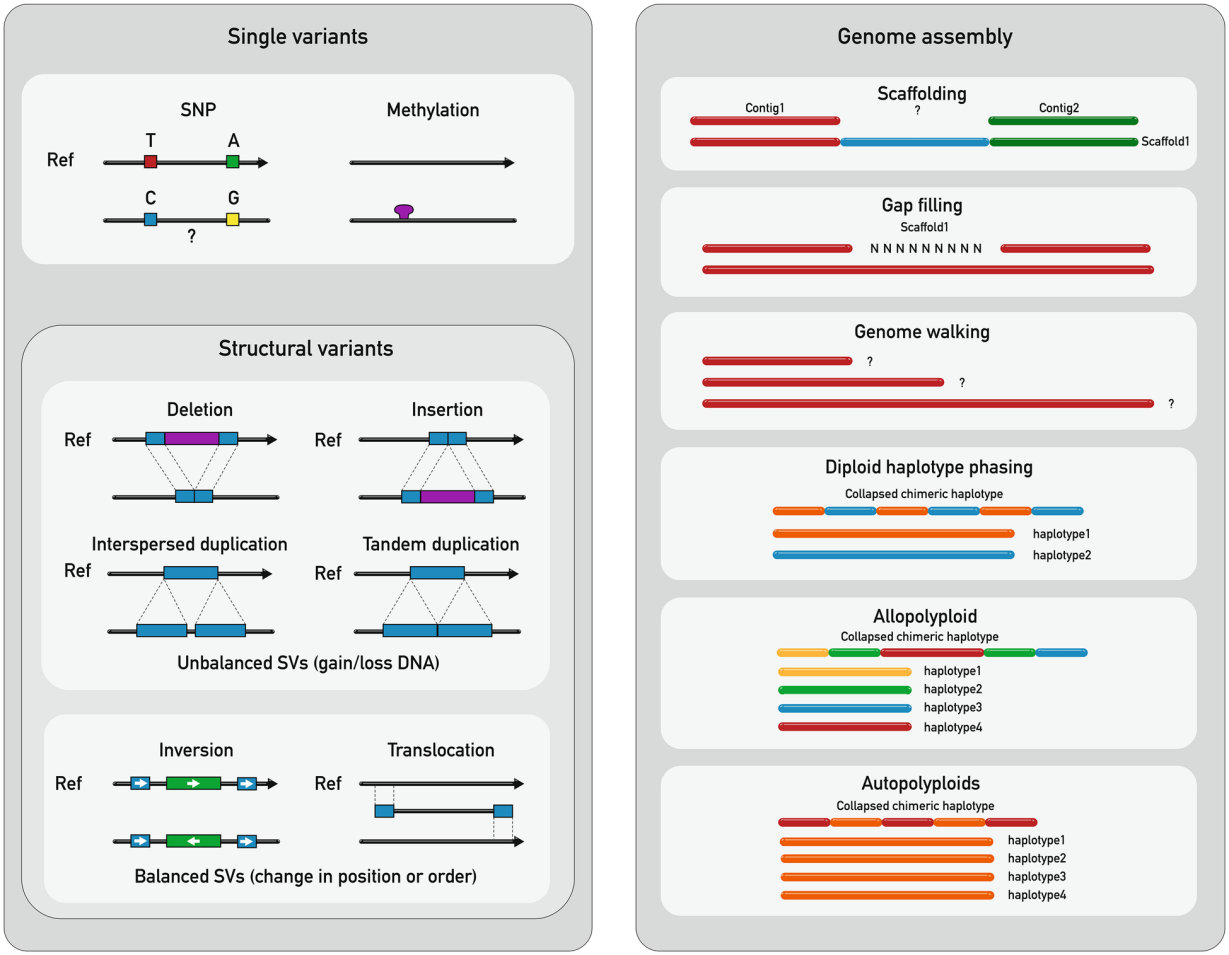
CRISPR-Cas9 targeted enrichment applications. The method can be used to identify single nucleotide genomic and epigenomic variants as well as long structural variants (SVs) (both, balanced and unbalanced SVs) by knowing one or both ends of the region of interest (ROI). It can improve genome assembly contiguity by assisting in the scaffolding and gap filling processes when two ends of the ROI are known or by genome walking approaches when just one side of the ROI is known. Phasing targeted loci of genome assemblies for both diploids and polyploid species is also possible with this technique

**Fig. 6:**
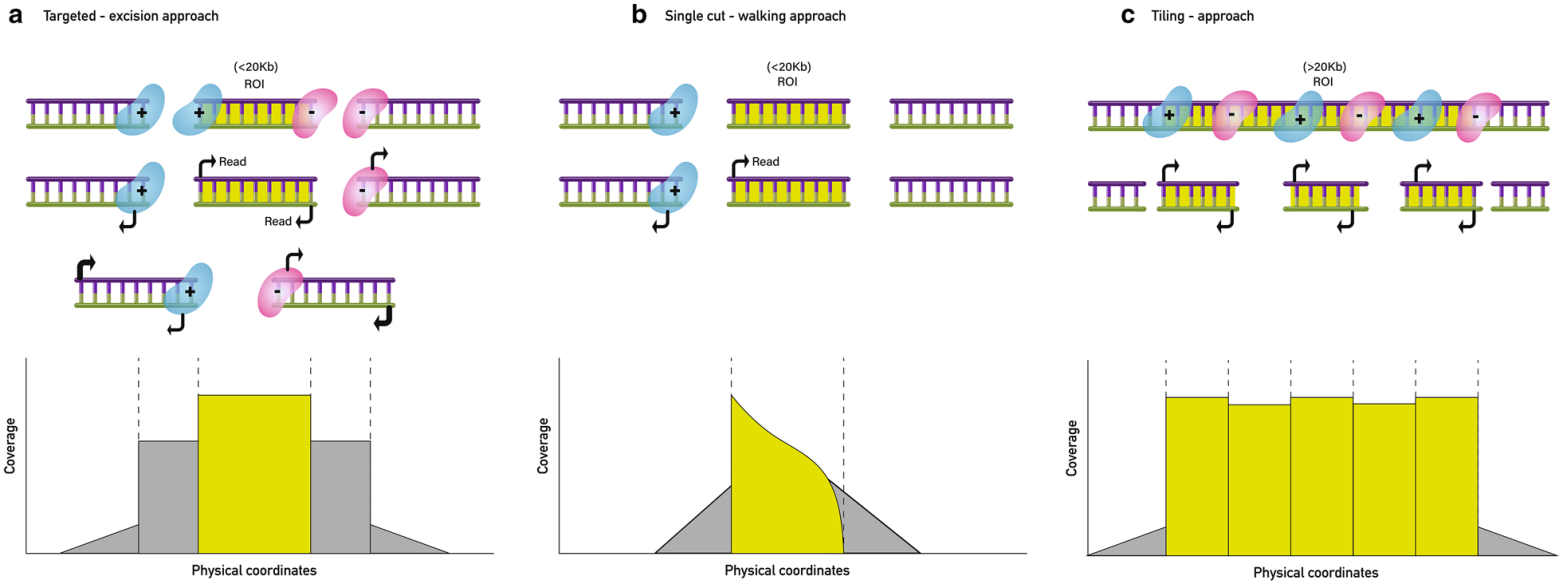
crRNA design approaches, adapted from Oxford Nanopore Technology (ONT). **a** Targeted-excision approach. The crRNAs are designed to either side of the region of interest (ROI). Both ends of the ROI must be known to be able to design the crRNAs. Indicated when the ROI is shorter than 20 kb (specific genes or genomic regions). **b** Single cut-walking. A single crRNA is designed at one known end of a ROI to walk towards the unknown. Indicated to target a ROI when just one side is known and for genome walking approaches of targeted regions shorter than 20 kb. **c** Tiling approach. Several crRNAs are designed across a ROI (which can be longer than 20 kb) in 5–10 kb overlapping pieces to get an even coverage along the region. Indicated especially when the length of the input DNA is limited

This method has the potential to be widely applied as a tool for identifying single nucleotide variants as a first step in developing trait-associated SNP-based markers for marker-assisted selection. It can also help characterize structural rearrangements, repetitive regions and methylation variants (Fig. 5). The design of crRNA probes to either side of an unknown sequence within a genome scaffold or the design of a single crRNA at one end of a target region (or the end of a scaffold) to read into the unknown could help in the scaffolding or genome walking process by closing sequencing gaps present in most published genome assemblies. A tiling approach could be applied for a deeper characterization of large regions of interest. However, some conserved or syntenic regions, from which crRNA probes could be designed targeting several smaller and overlapping fragments, would need to be known to obtain an even coverage across the region of interest (Fig. 6). In addition, our method could be employed to accurately detect in vitro off-targets mutations induced by Cas-RNP complexes before these are used in vivo for genome-editing purposes.

The major consideration for successfully using this sequence-capture approach is DNA quality.

Our preliminary evaluations found that total genomic DNA isolated using common CTAB extraction methods were unsuitable for CRISPR-Cas9 digestion and enrichment, while high molecular weight nuclear DNA was effective. Although, it involves a few more steps than standard total genomic DNA preparations, it is worth optimizing the protocol to avoid wasting valuable sequence data on organellar DNA. Furthermore, inhibitors affecting DNA digestion and reducing the sequencing of very long reads are removed using this protocol. The other critical aspect for success is efficient DNA digestion by RNP complexes in vitro. It is known that guide RNAs do not always work as efficiently as predicted [42, 43], so we evaluated the activity of RNP complexes to digest upon PCR amplicons. However, an alternative strategy could be to use several RNPs targeting a single region, either within the same digest, or separately, and then pooled for DNA enrichment and sequencing. This approach is likely to be particularly valuable if the CRISPR target sites have not been thoroughly sequenced (e.g. sequencing a locus from many samples), or when there are several haplotypes (e.g. isolating a locus from polyploid species).

Future improvements on our methodology will move into CRISPR-Cas9 multiplexing enrichment. The use of barcodes to index different samples enables a more efficient use of the ONT sequencing flow cells, increasing pore occupancy and therefore expanding the life of pores while sequencing.

We have shown that haplotype phasing is possible with this method due to the high-coverage ONT data. As the base-calling and variant-calling algorithms improve, we also anticipate a broader use of this methodology to phase targeted ONT sequencing reads into parental alleles as well as to determine dosage of specific alleles in polyploid species and diversity within breeding populations.

## Conclusion

We demonstrated the efficiency of combining an enriched CRISPR-Cas9 library with ONT sequencing for fine mapping the red flesh locus on the apple physical genome. This method could potentially be used for a broader range of trait and variant types, for example CpG methylation variants. Larger loci may be resolved using very long sequencing reads or by tiling the loci with multiple overlapping cleavage products. In future, several loci may also be multiplexed in one single run and multiplexed individual samples could be used employing barcoded adapters.

## Methods

### Plant material and DNA isolation

Intact and fully expanded but young leaves (20 g) were collected from a Type 1 red flesh *M.* × *domestica* accession (ABGS0131) grown at The New Zealand Institute for Plant and Food Research Limited, Havelock North (New Zealand) [33]. Nuclear genomic DNA was extracted as described at https://www.protocols.io/view/plant-nuclear-genomic-dna-preps-rncd5aw.

### Guide RNA design

Functional guide RNA duplexes were built from synthetic CRISPR RNAs (crRNAs; Integrated DNA Technologies (IDT), custom designed) and tracer RNA (tracrRNA, IDT, 1072532) to cut in complementary strands flanking the region of interest. The crRNA sequences (Table 1) were designed using Geneious’ 10.0.9 (https://www.geneious.com) ‘Find CRISPR sites’ tool using the apple reference genome (‘Golden Delicious’ double haploid GDDH13v1.1) [34] as a template for selecting the highest scored on-targets and avoiding off-targets. In addition, a blastn + [44] (command line mode “blastn-short”) search was performed (Additional file 1: Table S1) for the four selected crRNAs against the apple reference genome (‘Golden Delicious’ double haploid GDDH13v1.1) [34] to check the degree of sequence specificity.

### From gRNA complexes to Cas9 ribonucleoprotein complexes

All crRNAs were re-suspended and pooled in nuclease-free duplex buffer (IDT, 11010301) to a final concentration of 100 µM. Guide RNA complexes were built by adding equal amounts of pooled crRNAs and tracrRNA in duplex buffer to a final concentration of 10 µM and incubating for 5 min at 95 °C before the reaction was allowed to cool at room temperature for 5 min. Cas9 ribonucleoprotein complexes (RNPs) were assembled by mixing 10 pmol of gRNA duplexes with 10 pmol of Cas9 Nuclease V3 (IDT, 1081058) in 1 × CutSmart buffer (NEB, B7204) to a final volume of 30 µL. The complexes were incubated at room temperature for 30 min, then stored at −20 °C until use.

### Cas9 cleavage

The efficiency of Cas9 cleavage was verified by determining the absence of any variant on the crRNA sequences by PCR amplification and Sanger sequencing of cloned amplicons surrounding the region of interest. Primer sequences for amplifying crRNA amplicons can be found in Table 2. PCR reactions were carried out in a 50 µL volume containing 1 × PCR buffer mix (Invitrogen), 100 μM of each dNTP, 1.5 mM MgCl_2_, 0.5 μM each primer, 0.5 U Platinum™ DNA polymerase (Thermo Fisher Scientific, 10966034) and 20 ng template DNA. Amplifications were carried out on a MasterCycler ProS thermocycler (Eppendorf). The conditions of the touchdown PCR included an initial denaturing at 94 °C for 2 min, then five cycles (94 °C for 55 s, 60 °C for 55 s (decreased 1 °C each cycle), 72 °C for 1 min and 39 s), then 35 cycles (94 °C for 55 s, 55 °C for 55 s and 72 °C for 1 min and 39 s) and a final extension at 72 °C for 10 min. The Cas9 cleavage reactions were performed using 20 ng of amplicon DNA, 2 µL of Cas9-RNP complex specific to that amplicon, 2 µL of 10 × NEB buffer 3 and 12 µL of DNA-free water. Cleaved fragments of the PCR amplicons were separated by electrophoresis using a 5200 Fragment Analyzer (Advanced Analytical Technologies, Inc.).

A total of 5 µg of high molecular weight nuclear genomic DNA re-suspended in 30 µL of 1 × NEB CutSmart buffer (NEB, B7204) was dephosphorylated using 3 µL of Shrimp Alkaline Phosphatase (rSAP: NEB, M0371S) for 30 min at 37 °C, followed by a heat-inactivation step for 5 min at 65 °C and a cooling step to return the sample to room temperature. The dephosphorylated sample was mixed with 10 µL of pre-built pooled Cas9-RNP complexes, 1 µL of 10 mM dATP (Invitrogen, 10297-018) and 1 µL of Taq polymerase (NEB, M0267) and incubated for 30 min at 37 °C for Cas9 cleavage, followed by 5 min at 72 °C for A-tailing.

### Oxford Nanopore Technology sequencing library preparation

AMX adapters from ONT Ligation Sequencing Kit (SQL-LSK109) were ligated to the dA-tailed DNA ends using 20 µL of the following adapter mix: 10 µL of NEBNext Quick T4 DNA Ligase (NEB, M2200), 20 µL of Oxford Nanopore Ligation buffer (LNB, ONT, SQL-LSK109) and 3 µL of nuclease-free water. This reaction was incubated for 10 min at room temperature. The ligated sample was purified twice on a magnetic rack using 0.3 × volume of AMPure XP Beads (Becman Coulter, A63881) and 250 µL of long-fragment buffer (ONT, SQL-LSK109). Elution of the ligated sample was performed by adding 13 µL of elution buffer (ONT, SQL-LSK109), incubating for 10 min at room temperature and placing the tube back on the magnetic rack to collect 12 µL of the eluate. The Oxford Nanopore DNA sequencing library was then prepared by adding 25 µL of sequencing buffer (ONT, SQL-LSK109) and 13 µL of loading beads (ONT, SQL-LSK109) to the eluate. Flow cell priming mix was prepared by placing 30 µL of flush tether (ONT, SQL-LSK109) into a tube of flush buffer (ONT, SQL-LSK109).

### Oxford Nanopore sequencing

The sample library was run on a 9.4.1 version flow cell using an ONT MinION sequencer using MinKNOW software (ONT). Prior to loading the DNA library, the flow cell was primed by drawing back 230 µL of the buffer from the priming port followed by the addition of 200 µL of the priming mix. The DNA library was then added via the SpotON sample port in a dropwise fashion.

### Sequencing data analysis and alignment against the apple reference genome

Nanopore 9.4.1 raw FAST5 reads were base called using Albacore (v2.3.4, ONT) and Guppy (v3.2.4, ONT) and converted to FASTQ format. The quality of base-called sequencing reads was assessed using PycoQC (v2.2.3.3) [45]. Adapters were trimmed from read ends using Porechop (v0.2.3 https://github.com/rrwick/Porechop). Reads were corrected by using Canu’s (v1.7) [36] command ‘canu-correct’ and aligned to the apple reference genome (‘Golden Delicious’ double haploid GDDH13v1.1) [34] using Minimap2 (v2.9) [46]. Alignments were observed using the integrative genome viewer (IGV) [35]. Samtools (v1.9) [47] depth and bedtools (v2.27.1) [48] coverage were used to determine the per-nucleotide coverage. Coverage clustering was performed using the ‘bincov’ function from SURVIVOR (v1.0.7) [49] tool package and plotted using a custom-based Rmarkdown script found in https://github.com/PlantandFoodResearch/ONT_CRISPR-Cas9_enrichment.

### De novo assembly

The de novo assembly pipeline followed is shown in Fig. 1. The first draft de novo assembly was performed by the Canu (v1.7) assembler [36] using Albacore2 (v2.3.4, ONT) corrected reads as input. The command used was ‘nanopore-corrected’ with of 200 kb of genome length estimated based on the total length of loci that were aligned against the reference genome (‘Golden Delicious’ double haploid GDDH13v1.1) [34] and the possibility of getting de novo assembled loci. This draft assembly was further improved using Nanopolish (v0.11.1) software [37], which created an index to link raw reads with their signal-level data in the FAST5. Then, raw indexed reads were aligned to the draft assembly producing a consensus polished variant calling file (VCF) that was used to call true positive variants using a minor allele frequency of 10% to get the first Canu_nanopolished assembly. Subsequently, Canu_corrected reads and the Canu_nanopolished assembly were used as inputs to run the final assembly performed by Flye (v2.5) [38]. We evaluated the sequence and structural similarity of these assemblies using dnadiff wrapper for nucmer software [50].

### Characterization of haplotypes on enriched targeted loci and off-targets

Both Albacore2 and Guppy corrected reads were aligned by Minimap2 (v2.9) [46] against: (1) the Canu_nanopolished assembly to identify the contig containing the target locus; (2) Chr09 of the apple reference ‘Golden Delicious’ double haploid GDDH13v1.1 genome [34] to characterize the variants and mutations between the red-fleshed accession and ‘Golden Delicious’; and (3) Chr17 from apple reference genome GDDH13v1.1 [34] to ascertain whether the reads aligned to Chr09’s homologous chromosome.

## Supplementary information

**Additional file 1: Table S1.** Location of crRNAs sequences on the apple reference genome. Blastn+ hits of crRNAs in ‘Golden Delicious’ double haploid GDDH13v1.1 [34]. qseq: query sequence name; sseq:subject sequence name; pident: percentage of identical matches; qcovs: query coverage per subject; length: alignment length; mism: mismatches; gap: gap openings; qstart: start alignment in query; qend: end alignment query; sstart: start of alignment in subject; send: end of alignment in subject; sframe: subject frame; evalue: expected value; bitscore: bit score; qseq: aligned part of query sequence. Extended explanation of features at “Fassler CPJ. BLAST(R) help. Bethesda, MD: National Center for Biotechnology Information (US). https://www.ncbi.nlm.nih.gov/books/NBK62051/.”.

**Additional file 2: Figure S1.** Location of crRNAs sequences in relation to the apple ‘type 1’ red flesh locus and the MYB10 candidate gene. Pink arrows represent the location of crRNA_RF_1_F and crRNA_RF_3_R sequences on R1 (found in both white and red-fleshed apple varieties) and the R6 promoter (showing the repeat units found on the ‘Type 1’ red fleshed allele of the MYB10 gene) of the M. x domestica accession (ABGS0131) studied here, as previously shown in [33]. The vertical arrow indicates the ATG start side of the MYB10 gene.

**Additional file 3: Figure S2.** Pair-wise alignment between contig sequences generated by Canu assembler (v1.7) [36] and after polishing it by Nanopolish (v0.11.1) [37]. In pink is highlighted the protospacer adjacent motif (PAM) site and the 3 bp upstream of the PAM site of crRNA_RF_1_F where Cas9 performed the cleavage (^).The PAM site of crRNA_RF_3_R and one upstream bp before the cleavage site is highlighted in blue. In yellow is highlighted the sequence of crRNA_RF_2_F is highlighted in yellow. The nucleotide differences between both contigs are highlighted in green. The repeat units present in R1 and R6 promoter allele of MYB10 gene are highlighted in grey tones.

**Additional file 4: Figure S3.** Per base coverage plot of the alignment of canu-corrected reads against the de novo assembled canu-contig.

**Additional file 5: Figure S4.** Pair-wise alignment between haplotig A and haplotig B sequences generated by Flye assembler (v2.5) [38]. The protospacer adjacent motif (PAM) is site and the 3 bp upstream of the PAM site of crRNA_RF_1_F where Cas9 performed the cleavage (^) are highlighted in pink. The PAM site of crRNA_RF_3_R and one upstream bp before the cleavage site are highlighted in blue. The sequence of crRNA_RF_2_F is highlighted in yellow. The nucleotide differences between the contigs are highlighted in green. The repeat units present in R1 and R6 promoter allele of MYB10 gene are highlighted in grey tones. The sequences referred in line numbers preceded by (-) or (+) were not included in the de novo assembled contigs shown here.

## Data Availability

All data generated or analysed during this study are included in this published article and its additional information files. Sequencing data from the plant sample used in this study are available from SRA, under the BioProject ID PRJNA629607. The code and documentation used for the analysis of the data can be found in https://github.com/PlantandFoodResearch/ONT_CRISPR-Cas9_enrichment.
