# Supplementary material for: CRISPR-Cas9 enrichment and long read sequencing for fine mapping in plants
Source: Plant Methods. 2020 Sep 1;16:121. doi: 10.1186/s13007-020-00661-x (PMC7465313; doi:10.1186/s13007-020-00661-x)
Supplement: Supplementary file 1 — Additional file 1: Table S1. Location of crRNAs sequences on the apple reference genome. Blastn+ hits of crRNAs in ‘Golden Delicious’ double haploid GDDH13v1.1 [34]. qseq: query sequence name; sseq:subject sequence name; pident: percentage of identical matches; qcovs: query coverage per subject; length: alignment length; mism: mismatches; gap: gap openings; qstart: start alignment in query; qend: end alignment query; sstart: start of alignment in subject; send: end of alignment in subject; sframe: subject frame; evalue: expected value; bitscore: bit score; qseq: aligned part of query sequence. Extended explanation of features at “Fassler CPJ. BLAST(R) help. Bethesda, MD: National Center for Biotechnology Information (US). https://www.ncbi.nlm.nih.gov/books/NBK62051/.”. [file 13007_2020_661_MOESM1_ESM.docx]

**Additional file 1: Table S1.**

| **qseq-id** | **sseq-id** | **pident** | **qcovs** | **length** | **mism** | **gap** | **qstart** | **qend** | **sstart** | **send** | **sframe** | **evalue** | **bitscore** | **qseq** |
| --- | --- | --- | --- | --- | --- | --- | --- | --- | --- | --- | --- | --- | --- | --- |
| crRNA_RF_1_F | Chr09 | 100 | 100 | 23 | 0 | 0 | 1 | 23 | 35542701 | 35542723 | 1 | 6.7E-05 | 46.1 | GTCATATCTAAGGACCCGCGTGG |
| crRNA_RF_2_F | Chr09 | 100 | 100 | 23 | 0 | 0 | 1 | 23 | 35542848 | 35542870 | 1 | 6.7E-05 | 46.1 | TCTGTACTCCGTCTGTCGGTCGG |
| crRNA_RF_3_R | Chr09 | 100 | 100 | 23 | 0 | 0 | 1 | 23 | 35550711 | 35550689 | -1 | 6.7E-05 | 46.1 | AGAAGACTGTCAATCCCGAGTGG |
| crRNA_RF_3_R | Chr14 | 94.737 | 83 | 19 | 1 | 0 | 1 | 19 | 5006898 | 5006880 | -1 | 3.9 | 30.2 | AGAAGACTGTCAATCCCGA |
| crRNA_RF_4_F | Chr09 | 100 | 100 | 23 | 0 | 0 | 1 | 23 | 35551878 | 35551900 | 1 | 6.7E-05 | 46.1 | TGTCTGGAAAGTTTCTAACGCGG |
| crRNA_RF_4_F | Chr06 | 100 | 65 | 15 | 0 | 0 | 3 | 17 | 23658580 | 23658566 | -1 | 3.9 | 30.2 | TCTGGAAAGTTTCTA |
| crRNA_RF_4_F | Chr17 | 100 | 70 | 15 | 0 | 0 | 2 | 16 | 9145377 | 9145363 | -1 | 3.9 | 30.2 | GTCTGGAAAGTTTCT |
| crRNA_RF_4_F | Chr17 | 100 | 70 | 15 | 0 | 0 | 3 | 17 | 16542983 | 16542969 | -1 | 3.9 | 30.2 | TCTGGAAAGTTTCTA |
