# Supplementary figures and images for: CRISPR-Cas9 enrichment and long read sequencing for fine mapping in plants
Source: Plant Methods. 2020 Sep 1;16:121. doi: 10.1186/s13007-020-00661-x (PMC7465313; doi:10.1186/s13007-020-00661-x)

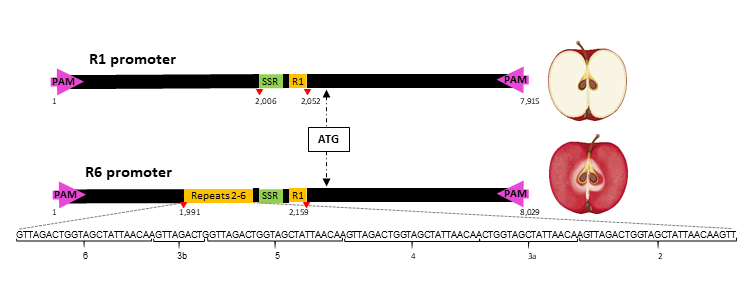

Supplement: Supplementary file 2 — Additional file 2: Figure S1. Location of crRNAs sequences in relation to the apple ‘type 1’ red flesh locus and the MYB10 candidate gene. Pink arrows represent the location of crRNA_RF_1_F and crRNA_RF_3_R sequences on R1 (found in both white and red-fleshed apple varieties) and the R6 promoter (showing the repeat units found on the ‘Type 1’ red fleshed allele of the MYB10 gene) of the M. x domestica accession (ABGS0131) studied here, as previously shown in [33]. The vertical arrow indicates the ATG start side of the MYB10 gene. [file 13007_2020_661_MOESM2_ESM.png]

**Additional file 4: Figure S3**


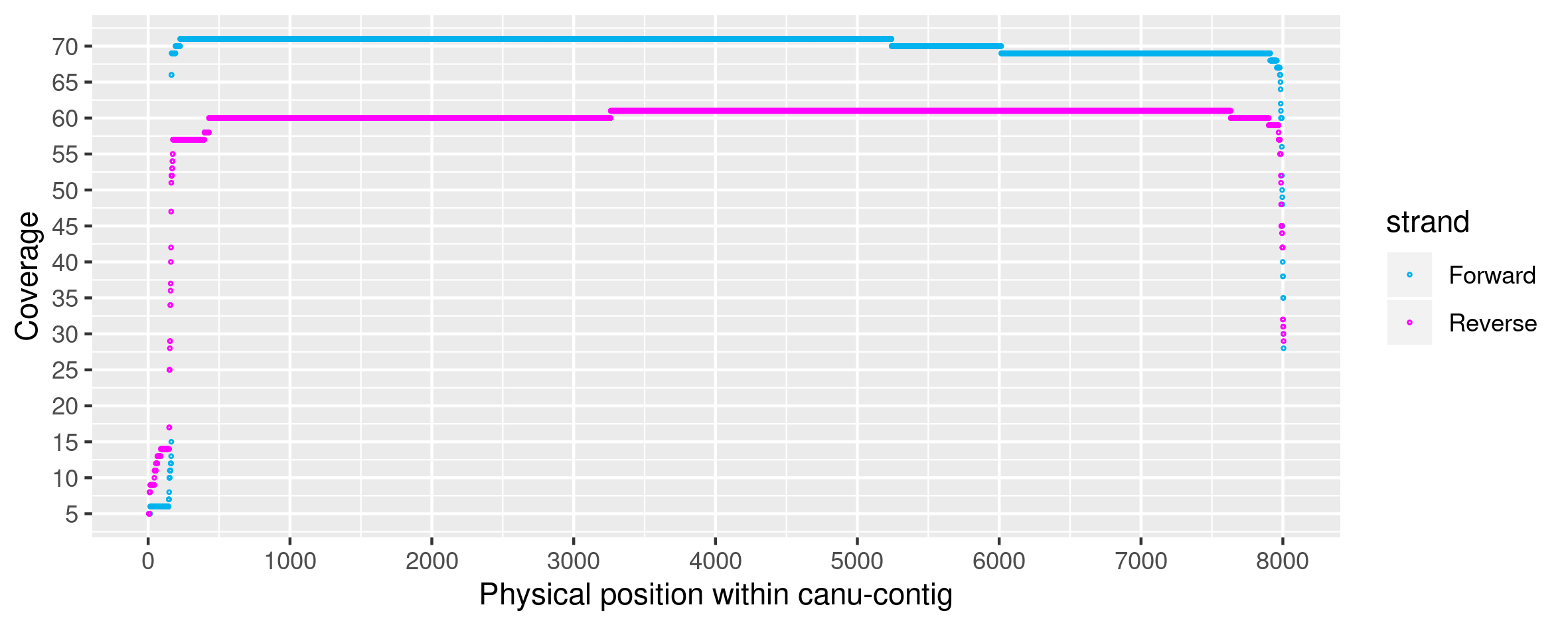

Supplement: Supplementary file 4 — Additional file 4: Figure S3. Per base coverage plot of the alignment of canu-corrected reads against the de novo assembled canu-contig. [file 13007_2020_661_MOESM4_ESM.docx]
