# Supplementary material for: CRISPR-Cas9 enrichment and long read sequencing for fine mapping in plants
Source: Plant Methods. 2020 Sep 1;16:121. doi: 10.1186/s13007-020-00661-x (PMC7465313; doi:10.1186/s13007-020-00661-x)
Supplement: Supplementary file 3 — Additional file 3: Figure S2. Pair-wise alignment between contig sequences generated by Canu assembler (v1.7) [36] and after polishing it by Nanopolish (v0.11.1) [37]. In pink is highlighted the protospacer adjacent motif (PAM) site and the 3 bp upstream of the PAM site of crRNA_RF_1_F where Cas9 performed the cleavage (^).The PAM site of crRNA_RF_3_R and one upstream bp before the cleavage site is highlighted in blue. In yellow is highlighted the sequence of crRNA_RF_2_F is highlighted in yellow. The nucleotide differences between both contigs are highlighted in green. The repeat units present in R1 and R6 promoter allele of MYB10 gene are highlighted in grey tones. [file 13007_2020_661_MOESM3_ESM.docx]

**Additional file 3: Figure S2**

-- BEGIN alignment [ +1 1 - 2008 | +1 1 - 2031 ]

1 t^gcgtggaaggggagcggagaggatgggctctcagcccggaccgaacca

1 t^gcgtggaaggggagcggagaggatgggctctcagcccggaccgaacca

**PAM**

50 tttccaccgttcatttctaagtttgaacaaatcctatctcaactatctg

50 tttccaccgttcatttctaagtttgaacaaatcctatctcaactatctg

99 ttgttgagttcgcaacggcaatgcaggcgcagatctgtactccgtctgt

99 ttgttgagttcgcaacggcaatgcaggcgcagatctgtactccgtctgt

148 cggtcagtctctcctatctcgaattccgaaaggcattgcctcttcatct

148 cggtcagtctctcctatctcgaattccgaaaggcattgcctcttcatct

197 ctctactgcagtgcctagaaatggttcatgtataacagtatcagttcgt

197 ctctactgcagtgcctagaaatggttcatgtataacagtatcagttcgt

246 gcatcagtggttcaattgcagtgctcagaaatcgttcgaaggtctaagg

246 gcatcagtggttcaattgcagtgctcagaaatcgttcgaaggtctaagg

295 tgacataaattccc..ctatttctgttcgaaatcttcaattctttagat

295 tgacataaattcccccctatttctgttcgaaatcttcaattctttagat

342 ttaaggtattcagttttagggatataggcttgaagaatcaattagggat

344 ttaaggtattcagttttagggatataggcttgaagaatcaattagggat

391 ttacaaaatgattaaagggattttgggtgtttgctgttgccatttttga

393 ttacaaaatgattaaagggattttgggtgtttgctgttgccatttttga

440 acacaacatcagttccactactctttcattttc.ctcaatttctgagca

442 acacaacatcagttccactactctttcattttccctcaatttctgagca

488 accaaacaagtagcattattgacaacatactgagctcctcgtgtcaacc

491 accaaacaagtagcattattgacaacatactgagctcctcgtgtcaacc

537 attcttgaaagaatcctaataaagatttataggcaaattatgccctaga

540 attcttgaaagaatcctaataaagatttataggcaaattatgccctaga

586 aaa.atttaat.aaaaggaccctgaacacgtaggaaccggcccgtttgt

589 aaaaatttaataaaaaggaccctgaacacgtaggaaccggcccgtttgt

633 aacagactgagataggtccggttctatttcttaaa.acccaacacccgc

638 aaccgactgagataggtccggttctatttcttaaaaacccaacacccgc

681 tacgttccatttataaacgggtcggtctggtccctccaactttgagccc

687 tacgttccatttataaacgggtcggtctggtccctccaactttgagccc

730 ggctcgacttgtgcccactcctaaactaaaccatataaa.accaagatt

736 ggctcgacttgtgcccactcctaaactaaaccatataaaaaccaagatt

778 tccc.tttcatctttcacacatatcacgttactttccaacaacaattca

785 tcccttttcatctttcacacatatcacgttactttccaacaacaattca

826 acaatcacaacaaataatcaaccatcaagatcatatatcacgtcactaa

834 acaatcacaacaaataatcaaccatcaagatcatatatcacgtcactaa

875 taaagacaaccttcataagggttgccgtagttctctacttgaaatccaa

883 taaagacaaccttcacaagggttgtcgtagttctctacttgaaatccaa

924 ttgtctagcattgtaaccctaagttacagacacaaacataaacttgagc

932 ttgtctagcattgtaaccctaagttacagacacaaacataaacttgagc

973 aacttctatgcataagaatctagggttttggactaactcaacagaacct

981 aacttctatgcataagaatctggggttttggactaactcaacagaacct

1022 aacaagaaataatattctggaccgcttaacggaatccaacgaagacaag

1030 aacaagaaataatattctggaccgcttaacggaatccaacgaagacaag

1071 gtttcggaccactcaacggaacaaataagggaaagggatataaaccatt

1079 gtttcggaccactcaacggaacaaataagggaaagggatataaaccatt

1120 caacgaaatccatctttagaatacgcatagt..ccccaatacggattaa

1128 caacgaaatccatctttagaatacgcatagtctccccaatacggattaa

1167 ccaagtgagaacatacgccatctgatagcgtggtcccgcaagacagtta

1177 ccaagtgagaacatacgccatctgatagcgtggtcccgcaagacagata

1216 accaagtaggaccaccgatggtataatgtgaccaagtaagcagtgaccc

1226 accaagtaggaccaccgatggtataatgtgaccaagtaagcagtgaccc

1265 taaatgtagattaaccacgtggagttaaattaacaaggctgaaccacct

1275 taaatgtagattaaccacgtggagttaaattaacaaggctgaaccacct

1314 atgaaaataatgtaagcctgaaatcttaggagagaattcttgctctagg

1324 atgaaaataatgtaagcctgaaatcttaggagagaattcttgctctagg

1363 ggacaaatgattttcgtatgcctaagtgtttt..tagtgacagtaaact

1373 ggacaaatgattttcgtatgcctaagtgtttttttagtgacagtaaact

1410 aagatttgagtacagagacattaactgagattgactcttgtgaaagctt

1422 aagatttgagtacagagacattaactgagattgactcttgtgaaagctt

1459 agtgagttgaagcacgtaggccaattatattgagcaatgtgttaggtgt

1471 agtgagttgaagcacgtaggccaattatattgagcaatgtgttaggtgt

1508 agcgtctaaacttccgtaggagttttgtacagcaatatagtggg.gtgc

1520 agcgtctaaacttccgtaggagttttgtacagcaatatagtgggggtgc

1556 cgcaaaatgcagacagtagcaataaattacgggctaggattttctcctc

1569 cgcaaaatgcagacagtagcaataaattacgggctaggattttctcctc

1605 ttttt...tcgttccattccatccattcctctcacattcttttattttg

1618 tttttttttcgttccattccatccattcctctcacatt.ttttattttg

1651 tctttctttc.tataaaaattaatataagatgttaatgtaacttgaccg

1666 tctttctttcata.aaaaattaatataagatgttaatgtaac.tgaccg

1699 cgactattcaaatagga.gggaatgaagaagagg..aaaaaagaggaga

1713 tgactattcaaataggaggggaatgaagaagagggaaaaaaaga.gaga

1745 gaatcctac.ccgtaaattacaagcaaacacttttttt......tggac

1761 gaatcctactccgtaaattacaagcaaacactttttttttttttttgac

1787 aagcagaagcaaacaaacacttgaaaaagcagcgaaagcatgataaagg

1810 aagcagaagcaaacaaacacttgaaaaagcagcgaaagcatgataaagg

1836 tatcttatggtggtcaaagatgtgtgttgtaactagttacacgattctg

1859 tatcttatggtggtcaaagatgtgtgttgtaactagttacacgattctg

1885 cattcacattcatagaatgtgcttttgaatattatattacagctagaga

1908 cattcacattcatagaatgtgcttttgaatattatattacagctagaga

1934 attttatgccctgggattgatttcccttgtcaatgttgtcgtgcagaaa

1957 attttatgccctgggattgatttcccttgtcaatgttgtcgtgcagaaa

1983 tgttagactggtagctattaacaagt

2006 tgttagactggtagctattaacaagt

6

95 bp INSERTION present in the canu-contig. Coordinates: 2006-2078

gttagactggttagactggtagctattaacaa

3b 5

gttagactggtagctattaacaactggtagctattaacaa

4 3a

-- END alignment [ +1 1 - 2008 | +1 1 - 2031 ]

-- BEGIN alignment [ +1 2079 - 8006 | +1 2007 - 7979 ]

2079 gttagactggtagctattaacaagttagactgtgtgtgtgtgtgtattt

2007 gttagactggtagctattaacaagt..g...gtgtgtgtgtgtgtattt

2 microsatellite

2128 cacaagttagactggtagctattaacaactgttggaatgttttaaactt

2051 cacaagttagactggtagctaataacaactgttggaatgttttaaactt

1

2177 gtcagtgtttgcttctgtggatatcagacatgcacgtcactggccttgt

2100 gtcagtgtttgcttctgtggatatcagacatgcacgtcactggccttgt

2226 aagattaattaggccgatggtatccatagcgttaacgtcatggcaaaca

2149 aagattaattaggccgatggtatccatagcgttaacgtcatggcaaaca

2275 cactctaattatatataatggtagctaggtgtctttctggagtctatga

2198 cactctaattatatataatggtagctaggtgtctttctggagtctatga

2324 agtgggtagcaggcaaaagataagctaagcttagctgctagcagataag

2247 agtgggtagcaggcaaagaataagctaagcttagctgctagcagataag

2373 agatggagggatataacgaaaacctgagtgtgagaaaaggtgcctggac

2296 agatggagggatataacgaaaacctgagtgtgagaaaaggtgcctggac

2422 tcgagaggaagacaatcttctcaggcagtgcgttgagattcatggagag

2345 tcgagaggaagacaatcttctcaggcagtgcgttgagattcatggagag

2471 ggaaagtggaaccaagtttcatacaaagcaggtatatatgttaatgtgt

2394 ggaaagtggaaccaagtttcatacaaagcaggtatatatgttaatgtgt

2520 atatttaactgtgaaagatggctatgtgtattattttaaagcatttcat

2443 atatttaactgtgaaagatggatatgtgtattattttaaagcatttcac

2569 tagtatttcattctaagaccttttgttaaatagtttcaagtttcaagtt

2492 tagtatttcattctaagaccttttgttaaatagtttcaagtttcaagtt

2618 ttacttttattaatgttttagaacatgttaatgtgtctaacggtcatac

2541 ttacttttattaatgttttagaacatgttaatgtgtctaacggtcatac

2667 ttgctctcacctcactcatctattgtgtttacatatatggctaaaatga

2590 ttgctctcacctcactcatctattgtgtttacatatatggctaaaatga

2716 cctatgcgtgtgtgag.gagggccatgttgagagacttagtccctcata

2639 cctatgcgtgtgtgagggagggccatgttgagagacttagtccctcata

2764 aatatttgttgttcacgtagaaagatgttatgtgaatgtaaactttgaa

2688 aatatttgttgttcacgtagaaagatgttatgtgaatgtaaactttgaa

2813 ttatgtatgcaggcttaaacaggtgcaggaagagctgcagacaaagatg

2737 ttatgtatgcaggcttaaacaggtgcaggaagagctgcagacaaagatg

2862 gttaaactatctgaagccaaatatcaagagaggagactttaaagaggat

2786 gttaaactatctgaagccaaatatcaagagaggagactttaaagaggat

2911 gaagtagatcttataattagacttcacaggcttttgggaaacaggtact

2835 gaagtagatcttataattagacttcacaggcttttgggaaacaggtact

2960 aataaataagtgtcattttcaattcatgtcgtcgttttcattgtacgga

2884 aataaataagtgtcattttcaattcatgtcgtcgttttcattgtacgga

3009 aattggacctattaacagtgagattataatcatagacctcaaactactt

2933 aattggacctattaacagtgagattataatcatagacctcaaactactt

3058 tttccactcttttaatattttaatgtttttcaatgaagtattagtggtg

2982 tttccactcttttaatattttaatgtttttcaatgaagtattagtggtg

3107 tgtagaat....aaaaaataaataaaaggtgtggtgtaagtaatttgaa

3031 tgtagaatataaaaaaaataaataaaaggtgttgtgtaagtaatttgga

3152 gtatgtgaatataatatttcttgttaatatattctggctccccatattt

3080 gtatgtgaatataatctttcttgttaatatattctggctccccatattt

3201 tcagtattttctaatacttcctaatttatatgtcattttatttttcatt

3129 tcagtattttctaatacttcctaatttatatgtcattttatttttcatt

3250 tagacatcaagcaaaaagttttcaattttgtagtattttt..tagattt

3178 tagacatcaagcaaaaagttttcaattttgtagtattttttttagattt

3297 att.aaaacaattatttcccaaattttt..tgtgggccaatggcctacc

3227 attaaaaacaattatttcccaaattttttttgtgggccaatggcctacc

3343 acatcattgtttaatggagaacttaaaggctagagtaacgaagtatgat

3276 acatcattgtttaatggagaacttaaaggctagagtaacgaagtatgat

3392 tttagagcaaatcgtaattttaggtataaaagtgagaggagaaaa.act

3325 tttagagcaaatcgtaattttaggtataaaagtgagaggagaaaaaact

3440 aagggtagcaacgtgcaaatttcacgatacttgagtatagtaaagtgag

3374 aagggtagcaacttgcaaatttcacgatacttgagtatagtaaagtgag

3489 gattactcttatttt.tagctatagtctagcatgagaatctaaactaca

3423 gattactcttattttttagctatagtctagcatgagaatctaaactaca

3537 aaatcattagagagggcaagcgttataaacattcattttaaatttt.ta

3472 aaatcattagagagggcaagcgttataaacattcattttaaatttttta

3585 atattataatattctaccttaagg.gcagagttg.tttttggttaagca

3521 atattataatattctaccttaaggggcagagttgttttttggttaagca

3632 aaacaaaa.atcattcgtatcaaatgtggtatcaatgaaaatcaaactt

3570 aaacaaaaaatcattcgtatcaaatgtggtatcaatgaaaatcaaactt

3680 cagactttagtcttaatttttaaaatgaagacaaatatcgagtgctaat

3619 cagactttagtcttaatttttaaaatgaagacaaatatcgagtgctaat

3729 agca....accaaaaattttaggaactgtttgatatcttatttgaaa.t

3668 agcaataaaccaaaaattttaggaactgtttggtatcttatttgaaatt

3773 ttttatcatttctctaaacatttcttaaaaacatttcttgaaaacaatt

3717 ttttatcatttctcaaaacattt.ttaaaaacatttcttgaaaacaatt

3822 ttctttaagacgcaaaaacttgatgagtatgcaaattaaaaatttcaaa

3765 ttctttaagactcaaaaacttgatgggtatgcaaattaaaaatttcaaa

3871 tcttacgactttaactagacaaagagatctaaacagaggg.gtcacggt

3814 tcttacgactttaactagacaaagagatctaaacagagggggtcacggt

3919 agagggagaaagaagataggaggaaaaaaagtaagagatgattgaaaga

3863 agagggagaaagaagataggaggaaaaaaagtaagagatgattgaaaga

3968 aagaaaaataagagagatgatccgatgagatagagaggaatatgagtga

3912 aagaaaaataagagagatgatccgatgagatagagaggaatatgagtga

4017 gagaaagaaccgagaggataaaaagagcagattggagaaaagacgaaaa

3961 gagaaagaaccgagaggataaaaagagcggattggagaaaagacgaaaa

4066 aggta....aaaaaaaaggagagagaaagaagaaaagagagagatagat

4010 aggtaataaaaaaaaaaggagagagaaagaagaaaagagagagatagat

4111 ttggagagagagaggagg.aggagag.aaaagaaataagagattttaag

4059 ttggagagagagaggaggaagaagagaaaaataaataagagagtttaag

4158 tttaaaaactctaaaactcacttt.tatgtttttagataatagactata

4108 tttaaaaactctaaaactcactttttatgtttttagataatagactata

4206 ttttt.agttagtcttgagttcaattt.taaaaatagtcctaccaaaca

4157 tttttgagttagtcttgagttcaatttttaaaaatagtcctaccaaaaa

4253 ag.ttttaaggcctaaaacttgaaaattgtttttgagtttaaa.agttg

4206 agtttttaaggcctaaaacttgaaaattgtttttgagtttaaaaagttg

4300 gattcaaataaagtaccaaacaagtccttagtttt.tcttgaccgaaaa

4255 gattcaaataaagtatcaaacaagtccttagttttttcttgaccgaaaa

4348 aataaaaatctttatccggaagggcattagtaaactcaaacaacctttc

4304 aataaaaatctttatccggaagggcattagtaaactcaaacaacctttc

4397 ttcgtaatgatttttgtatgtaaagtcattttcatgcttttaatcccta

4353 ttcgtaatgatttttgtatgtaaagtcattttcatgcttttaatcccta

4446 gtcgactgagcaaacctttaagattgtgtattcggcctacagaggcttg

4402 gtcgactgagcaaacctttaagattgtgtattcggcctacagaggcttg

4495 gatcagaatagataagaagttatacattcaaaatttcacaattaataaa

4451 gatcagaatagataagaagttatacattcaaaatttcacaattaataga

4544 attagaagggagaactttggtaataaatacacgcagtaattttatttt.

4500 attagaagggagaactttggtaataaatacacgcagtaattttattttt

4592 tgtattaaaactaatgttcgggcaaggatttggcctttgcacagctccc

4549 tgtattaaaactaatgttcgggcaaggatttggcctttgcacagctccc

4641 ttggagtgttggcacttggtgttgatgttggttgttggtcgagttcttg

4598 ttggagtgttggcacttggcgttgatgttggttgttggtcgagttcttg

4690 ctacatggtgtgctacaagaagagtacaaagttagttttgattgtgcct

4647 ctacttggtgtgctacaagaagagtacaaagttagttttgattgtgcct

4739 ttgtggggctttagatgtaggtcttgaggctcacaatcaaaactaacaa

4696 ttgtggggctttagatgtaggtcttgaggctcacaatcaaaactaacaa

4788 agagttaggcgt.ccactgttatctcaatataatagatgttgaatatat

4745 aaagttaggcgtgccactgttatctcaatataatagatgttgaatatat

4836 ttgatctaagtcgattacttgctctaagatgcttaaacttcttgttatc

4794 ttgatctaagtcgattacttgctctaagatgcttaaacttcttgttatc

4885 aaaggattgtcacaaatgggttaagtctgtattaagttctttt.tcttg

4843 aaaggattgtcacaaatgggttaagtctgtattaagttcttttttcttg

4933 ccttgtgaccaaggactttttcttatagttttgtatgtttagatgaag.

4892 ccttgtgaccaaggactttttcttatagttttgtatgtttagatgaagg

4981 gagtcc.gaacccttttaatcattcgcttgattacaattcgacgcttaa

4941 gagtccataacccttttaatcattcgcttgattacaattcgacgcttaa

5029 agaagtgaagttaacttgcttagccaaatttgatcataaatgggtctta

4990 agaagtgaagttaacttgcttagccaaatttgatcataaatgggtctta

5078 aagcgagaaacaattgcaagtgagttaaaacataacagaatatgcattt

5039 gagcgagaaacaattgtaagtgagttaaaacataacagaatatgcattt

5127 aaacaacagatctacaaactgtaagtacaaacacaaggaagttgggcaa

5088 aaacaacaaatctacaaactgtaagtacaaacacaaggaagttgggcaa

5176 gatttcaccttgtcgggcaaagttcaaacctcttgcaaccacttctctt

5137 gatttcaccttgtcgggcaaagttcaaacctcttgcaaccacttctcct

5225 tgagtttgtagaagagttgtgggtattgcaaaatggaaatgtaagcaag

5186 tgagtttgtagaagagttgtgggtattgcaaaatggaaatgtaagcaag

5274 aaatacaaataaggtttcctaaagggaatctattctacgaatctaggat

5235 aaatacaaacaaggtttcctaaagggaatctattctacgaatctaggat

5323 aaggtagcagagctttgccaaaagatgactttctgcgtggaatctatgg

5284 aaggtagcagagctttgccaaaagatgactttctgcgtggaatctatgg

5372 ctaaaaggtgcagaatctggacaaagtgcagc.ttttgggtatttgctt

5333 ctaaaaggtgcagaatctggacgaagtgcagctttttggttatttgctt

5420 gtctgagaggcaagtttgtatgtcttttgtttgattggttgagtgtcct

5382 gtctgagaggcaagtttgtatgtcttttgtttgattggttgagtgtcct

5469 .tgtctct.ttgtctctttctccttttatagacgatttggcccgactgc

5431 ttgtctctgttgtctctttctccttttatagacgatttggcccgactgc

5516 ttttagctctattcttgtccgaaagctcttggagggcaatgagtcatca

5480 ttttggctctattcttgtccgaaagctcttggagggcaatgagtcatca

5565 tcttttacttgtagtgccattagaaagtgttttttggctaatagtgagt

5529 tcttttacttgtagtgccattagaaagtgttttttggctaatagtgagt

5614 tggtctctgtcacttgtcacttcactcctacacatgtgtggcctatatt

5578 tgatctctgtcacttgtcacttcactcctacacatgt..ggcctatatt

5663 ttaattgaggaggcaccaatttgttacaggcttgtcgactgggcctcgg

5625 ttaattgaggaggcaccaatttgttacaggcttgtcgactgggcctcgg

5712 gcaagtcttcacttaatttgacatccatgggccttgactatttacaaaa

5674 gcaagtcttcacttaatttgacatccatgggtcttgactatttacaaaa

5761 ccctatgttaaatattaactcaaacaactagtccactccatttaattct

5723 ccttatgttaaatattaactcaaacaactagtccactccatttaattct

5810 aaagaagaaaatcgtttatgcaatctctgttc..ttttttttctttatt

5772 aaagaagaaaatcgtttatgcaatctctgttcctttttttttctttatt

5857 catcttatttttcaggcaaatgtattcatcattttttcttcatgcatgt

5821 catcttatttttcaggcaaatgtattcatcattttttcttcatgcatgt

5906 aatgaacttaggtggtcattgattgctagaagacttccaggaagaacag

5870 aatgaacttaggtggtcattgattgctagaagacttccaggaagaacag

5955 caaatgctgtgaaaaattattggaacactcgattgcggatcgattctcg

5919 caaatgctgtgaaaaattattggaacactcgattgcggatcgattctcg

6004 catgaaaacggtgaaaaataaatctcaagaaatgagagagaccaatgtg

5968 catgaaaacggtgaaaaataaatctcaagaaatgagaaagaccaatgtg

6053 ataagacctcagccccaaaaattcaacagaagttcatattacttaagca

6017 ataagacctcagccccaaaaattcaacagaagttcatattacttaagca

6102 gtaaagaaccaattctagaccatattcaatcagcagaagatttaagtac

6066 gtaaagaaccaattctagaccatattcaatcagcagaagatttaagtac

6151 gccaccacaaacgtcgtcgtcaacaaagaatggaaatgattggtgggag

6115 gccaccacaaacgtcgtcgtcaacaaagaatggaaatgattggtgggag

6200 accttgttagaaggtgaggatacttttgaaagagctgcatatcccagca

6164 accttgttagaaggcgaggatacttttgaaagagctgcatatcccagca

6249 ttgagttagaggaagaactcttcacaagtttttggtttgatgatcgact

6213 ttgagttagaggaagaactcttcacaagtttttggtttgatgatcgact

6298 gtcgccaagatcatgcgccaattttcctgaaggacaaagtagaagtgaa

6262 gtcgccaagatcatgcgccaattttcctgaaggacaaagtagaagtgaa

6347 ttctcctttagcacggacctttggaatcattcaaaagaagaatagctag

6311 ttctcctttagcacggacctttggaatcattcaaaagaagaatagctag

6396 agaaaatgattctcacttctgtagtatcatctagcttgtgtactattat

6360 agaaaatgattctcacttctgtagtatcatctagcttgtgttctattat

6445 tttccttgcttgtaaatgtggcatgtaaatatcattaagcttgatgaaa

6409 tttccttgcttgtaaatgtggcatgtaaatatcattaagcttgatgaaa

6494 ttgagattccaccataaaaccaattggaaatatggagagtagcccaaga

6458 ttgagattccaccataaaaccaattggaaatatggggagtagcccaaga

6543 ccatataagcacatagcaaaccttgtc.ctcaccgatgtggtacaaccg

6507 ccatataagcacatagcaaaccttgtccctcaccgatgtggtacaactg

6591 tcaacaca.ccctcgcatgtgtggcagattttcaagcctacacgtggat

6556 tcaacacacccctcgcatgtgtggcagattttcaagcctacacgtggac

6639 aacaactgggtgacgtggagcgatgtggccatttggcttcacacgagga

6605 aacaaccgggtgacgtggagcgatgtggccatttggcttcacacgagga

6688 caacccgctctaataccatgatgaaattaaggatccaccgtaaaactaa

6654 caacccgctctaataccatgatgaaattaaggatccaccgtaaaaccaa

6737 ttggtaatatgaggagtagcctaagaccatataagcacatagcaaacct

6703 ttggtaatatggggagtaggctaagaccatataagcacatagcaaacct

6786 tgttcctcaccgatatgggacaactgtcaacaaagctgacgaggaatcc

6752 tgtccctcaccgatatggaacaactgtcaacaaagctgacgaggaatcc

6835 taatcactggacttattttaataagtgaactatttttaatagtggttca

6801 taatcactggacttattttaataagtgaactatttttaatagtggttca

6884 caaaattggacttaaatcaaattttctcaaaataaattcagcaactacc

6850 caaaattggacttaaatcaaattttctcaaaataaattcagcaactacc

6933 acaaccaagttctttcatgtacaaatgtggctgaatcatataagcaata

6899 acaaccaagttctttcatgtacaaatgtggctgaatcatataagcaata

6982 aa.actaacaacttaaaggaaattctatacttgtaatcttctgcatttt

6948 aaaactaacaacttaaaggaaattctatacttgtaatcttctgcatttt

7030 caaagattccaaaggtcctccataaaggagaagcccctgccactcctta

6997 caaagattccaaaggtcctccataaaggagaagcccctgccactcctta

7079 ccttcatcaaaactgccacatgatcttgttaattggaccctagacttca

7046 ccttcatcaaaactgccacatgatcttgttaattggaccctagacttca

7128 acccaaaagtctgaatggatctttcatgtaatccaagactagaaattgt

7095 acccaaaagtctgaatggatctttcatgtaatccaagactagaaattgt

7177 tgttctgtgaagtacactatcggggatctcatttccaacaaccagtttg

7144 tgttctgtgaagtacactatcggggatctcatttccaacaaccagtttg

7226 attctctattggatgcgatcatccttgca...tttttactacaatggtt

7193 attctctattggatgcgatcatccttgcaatttttttgctacaatggtt

7272 ttctgattaattttgttctgcaatcaccacttctcaaacgcagtgaact

7242 ttctgattaattttgttctgcaatcaccacttctcaaacgcagtgaact

7321 atatattgaagaaggtttgtctaaagaaaa.atggaagtgtttccacga

7291 atatattgaagaaggtttgtctaaagaaaaaatggaagtgtttccacga

7369 gaaagtgtagacaaaatcatgtctatgaacaacagttccaattaaagaa

7340 gaaagtgtagacaaaatcaagtctatgaacaacagttccaattgaagaa

7418 aatcatctagtctcagaggttgcggcgttacaatgctccaaatgcatac

7389 aatcatctactctcagaggttgtggcgttacaatgctccaaatgcatac

7467 gataatgcaaaatcatgggattaatttcacagaaattgtagacaaaatc

7438 gataatgcaaaatcatgggattaatttcacagaaattgtagacaaaatc

7516 acataatctacaaacccctagagccaggaacatcaatccactgtaattg

7487 acataatctacaaacccctagagccaggaacatcaatccactgtaattg

7565 gagctccagttccccactctccacatttctcagcctgatacacatattc

7536 gagctccagttccccactctccacatttctcagcctgatacacatattc

7614 tggacaacttggccatcagtatggacgatgcagctctcttcagctagac

7585 tggacaacttggccatcagtatagacgatgcagctctcttcagctagac

7663 agttttgccggcttggttggaatcttgaaattatggttccacttggtac

7634 agttttgccggcttggttggaatcttgaaattatggttccacttggtac

7712 gccttgcaaacccacacgtaaagcttgaacaaaaggaccaatctcaaac

7683 gccttgcaaacccacacgtagagcttgaacaaaaggaccaatctcaaac

7761 tcagcatccc.catcttgtcatcaagactaaacgtgtctttgtcataca

7732 tcagcatcccccatcttgtcatcaagactaaacgtgtctttgtcataca

7809 caaactgcaatataagcaccccaaaa.attcagaacaatgtccaaagtt

7781 caaactgcaatataagcaccccaaaaaattcagaacaatgtccgaagtt

7857 tcatttcatgcctcacacaggccgaaaaatctcaggaccagccctgtat

7830 tcatttcatgcctcacacaggccgaaaaatctcaggaccagccctgtat

7906 gcgaaagacaatcaatccagaatgtttgatagaaaatagtacttacaag

7879 gcgaaagacaatcaatccagaatgtttgatagaaaatagtacttacaag

7955 tttgataggaaggcttggatctgcaaccgaaagagttaagtcttcgttc

7928 tttgataggaaggcttggatctgcaaccgaaagagttaagtcttcgttc

8004 cac^

7977 cac^

**PAM**

-- END alignment [ +1 2079 - 8006 | +1 2007 - 7979 ]
