# Supplementary material for: CRISPR-Cas9 enrichment and long read sequencing for fine mapping in plants
Source: Plant Methods. 2020 Sep 1;16:121. doi: 10.1186/s13007-020-00661-x (PMC7465313; doi:10.1186/s13007-020-00661-x)
Supplement: Supplementary file 5 — Additional file 5: Figure S4. Pair-wise alignment between haplotig A and haplotig B sequences generated by Flye assembler (v2.5) [38]. The protospacer adjacent motif (PAM) is site and the 3 bp upstream of the PAM site of crRNA_RF_1_F where Cas9 performed the cleavage (^) are highlighted in pink. The PAM site of crRNA_RF_3_R and one upstream bp before the cleavage site are highlighted in blue. The sequence of crRNA_RF_2_F is highlighted in yellow. The nucleotide differences between the contigs are highlighted in green. The repeat units present in R1 and R6 promoter allele of MYB10 gene are highlighted in grey tones. The sequences referred in line numbers preceded by (-) or (+) were not included in the de novo assembled contigs shown here. [file 13007_2020_661_MOESM5_ESM.docx]

**Additional file 5: Figure S4**

-- BEGIN alignment [ +1 1 - 1991 | +1 5 - 1986 ]

-8 t^gcgtgga

**PAM**

1 aggggagcggagaggatgggctctcagcccggaccgaaccatttccacc

5 aggggagcggagaggatgggctctcagcccggaccgaaccatttccacc

50 gttcatttctaagtttgaacaaatcctatctcaactatctgttgttgag

54 gttcatttctaagtttgaacaaatcctatctcaactatctgttgttgag

99 ttcgcaacggcaatgcaggcgcagatctgtactccgtctgtcggtcagt

103 ttcgcaacggcaatgcaggcgcagatctgtactccgtctgtcggtcagt

148 ctctcctatctcgaattccgaaaggcattgcctcttcatctctctactg

152 ctctcctatctcgaattccgaaaggcattgcctcttcatctctctactg

197 cagtgcctagaaatggttcatgtataacagtatcagttcgtgcatcagt

201 cagtgcctagaaatggttcatgtataacagtatcagttcgtgcatcagt

246 ggttcaattgcagtgctcagaaatcgttcgaaggtctaaggtgacataa

250 ggttcaattgcagtgctcagaaatcgttcgaaggtctaaggtgacataa

295 attccccctatttctgttcgaaatcttcaattctttagatttaaggtat

299 attccccctatttctgttcgaaatcttcaattctttagatttaaggtat

344 tcagttttagggatataggcttgaagaatcaattagggatttacaaaat

348 tcagttttagggatataggcttgaagaatcaattagggatttacaaaat

393 gattaaagggattttgggtgtttgctgttgccatttttgaacacaacat

397 gattaaagggattttgggtgtttgctgttgccatttttgaacacaacat

442 cagttccactactctttcattttccctcaatttctgagcaaccaaacaa

446 cagttccactactctttcattttccctcaatttctgagcaaccaaacaa

491 gtagcattattgacaacatactgagctcctcgtgtcaaccattcttgaa

495 gtagcattattgacaacatactgagctcctcgtgtcaaccattcttgaa

540 agaatcctaataaagatttataggcaaattatgccctagaaaaatttaa

544 agaatcctaataaagatttataggcaaattatgccctagaaaaatttaa

589 taaaaaggaccctgaacacgtaggaaccggcccgtttgtaacagactga

593 taaaaaggaccctgaacacgtgggaaccggcccgtttgtaaccgactga

638 gataggtccggttctatttcttaaaacccaacacccgctacgttccatt

642 gataggtccggttctatttcttaaaacccaacacccgctatgttctatt

687 tataaacgggt.cggtctggtccctccaactttgagcccggctcgactt

691 tataaacgggtccggtctggtccctccaactttgagcccggctcgactt

735 gtgcccactcctaaactaaaccatataaaaaccaagatttccctt.tca

740 gtgcccactcctaaactaaaccatataaaaaccaagatttcccttttct

783 tctttcacacatatcacgttactttccaacaacaattcaacaatcacaa

789 tctttcacacatatcacgttactttccaacaacaattcaacaatcacaa

832 caaataatcaaccatcaagatcatatatcacgtcactaataaagacaac

838 caaataatcaaccatcaagatcatatatcacgtcactaataaagacaac

881 cttcataagggttgccgtagttctctacttgaaatccaattgtctagca

887 cttcacaagggttgtcgtagttctctactggaaatccaattgtctagca

930 ttgtaaccctaagttacagacacaaacataaacttgagcaacttctatg

936 ttgtaaccctaagttacagacacaaacataaacttgagcaacttctatg

979 cataagaatctagggttttggactaactcaacagaacctaacaagaaat

985 cataagaatctggggttttggactaactcaacagaacctaacaagaaat

1028 aatattctggaccgcttaacggaatccaacgaagacaaggtttcggacc

1034 aatattctggaccgcttaacggaatccaacgaagacaaggtttcggacc

1077 actcaacggaacaaataagggaaagggatataaaccattcaacgaaatc

1083 actcaacggaacaaataagggaaagggatataaaccattcaacgaaatc

1126 catctttagaatacgcatagtcccccaatacggattaaccaagtgagaa

1132 catctttagaatacgcatagtctcccaatacggattaaccaagtgagaa

1175 catacgccatctgatagcgtggtcccgcaagacagttaaccaagtagga

1181 catacgccatctgatagcgtggtcccgcaagacagataaccaagtagga

1224 ccaccgatggtataatgtgaccaagtaagcagtgaccctaaatgtagat

1230 ccaccgatggtataatgtgaccaagtaagcagtgaccctaaatgtagat

1273 taaccacgtggagttaaattaacaaggctgaaccacctatgaaaataat

1279 taaccacatggagttaaattaacaaggctgaaccacctatgaaaataat

1322 gtaagcctgaaatcttaggagagaattcttgctctaggggacaaatgat

1328 gtaagcctgaaatcttaggagagaattcttgctctaggggacaaatgat

1371 tttcgtatgcctaagtgttttttagtgacagtaaactaagatttgagta

1377 tttcgtatgcctaagtgttttttagtgacagtaaactaagatttgagta

1420 cagagacattaactgagattgactcttgtgaaagcttagtgagttgaag

1426 cagagacattaactgagattgactcttgtgaaagcttagtgagttgaag

1469 cacgtaggccaattatattgagcaatgtgttaggtgtagcgtctaaact

1475 cacgtaggccaattatattgagcaatgtgttaggtgtagcgtctaaact

1518 tccgtaggagttttgtacagcaatatagtgggggtgccgcaaaatgcag

1524 tccgtaggagttttgtacagcaatatagtgggggtgccgcaaaatgcag

1567 acagtagcaataaattacgggctaggattttctcctctttttttcgttc

1573 acagtagcaataaattacgggctaggattttctcctctttttttcgttc

1616 cattccatccattcctctcacattctttattttgtctttctttctataa

1622 cattccatccattcctctcacattttttattttgtctttctttctataa

1665 aaaattaatataagatgttaatgtaacttgaccgtgactattcaaatag

1671 aaaattaatataagatgttaatgtaacttgaccgtgactattcaaatag

1714 gagg.gaatgaagaagaggaaaaagagaggagagaatcctactccgtaa

1720 gaggggaatgaagaagaggaaaaa...aggagagaatcctactccataa

1762 attacaagcaaacactttttttttggacaagcagaagcaaacaaacact

1766 attacaagcaaacact.........gacaagcagaagcaaacaaacact

1811 tgaaaaagcagcgaaagcatgataaaggtatcttatggtggtcaaagat

1806 tgaaaaagcagcgaaagcatgataaaggtatcttatggtggtcaaagat

1860 gtgtgttgtaactagttacacgattctgcattcacattcatagaatgtg

1855 gtgtgttgtaactagttacacgattctgcattcacattcatagaatgtg

1909 cttttgaatattatattacagctagagaattttatgccctgggattgat

1904 cttttgaatattatattacagctagagaattttatgccctgggattgat

1958 ttcccttgtcaatgttgtcgtgcagaaatgttag

1953 ttcccttgtcaatgttgtcgtgcagaaatgttag

>Haplotig A_rc: 1992-2111

actggtagctattaacaagttagactggttagactggtagctattaacaa

6 3b 5

gttagactggtagctattaacaactggtagctattaacaagttagactggtagctattaacaagtt

4 3a 2

agac

>Haplotig B_rc: 1987-2006

CTTTTCTATATATCGAGTGT

-- END alignment [ +1 1 - 1991 | +1 5 - 1986 ]

-- BEGIN alignment [ +1 2112 - 8026 | +1 2006 - 7915 ]

2112 tgtgtgtgtgtgtgtatttcacaagttagactggtagctattaacaact

2006 tgtgtgtgtgtgtgtatttcacaagttagactggtagctaataacaact

microsatellite 1

2161 gttggaatgttttaaacttgtcagtgtttgcttctgtggatatcagaca

2055 gttggaatgttttaaacttgtcagtgtttgcttctgtggatatcagaca

2210 tgcacgtcactggccttgtaagattaattaggccgatggtatccatagc

2104 tgcacgtcactggccttgtaagattaattaggccgatggtatccatagc

2259 gttaacgtcatggcaaacacactctaattatatataatggtagctaggt

2153 gttaatgtcatggcaaacacactctaattatatataatggtagctaggt

2308 gtctttctggagtctatgaagtgggtagcaggcaaaagataagctaagc

2202 gtctttctggagtgtatgaagtgggtagcaggcaaaagaatagctaagc

2357 ttagctgctagcagataagagatggagggatataacgaaaacctgagtg

2251 ttagctgctagcagataagagatggagggatataacgaaaacctgagtg

2406 tgagaaaaggtgcctggactcgagaggaagacaatcttctcaggcagtg

2300 tgagaaaaggtgcctggactcgagaggaagacaatcttctcaggcagtg

2455 cgttgagattcatggagagggaaagtggaaccaagtttcatacaaagca

2349 cgttgagattcatggagagggaaagtggaaccaagtttcatacaaagca

2504 ggtatatatgttaatgtgtatatttaactgtgaaagatggctatgtgta

2398 ggtatatatgttaatgtgtatatttaactgtgaaagatggatatgtgta

2553 ttattttaaagcatttcattagtatttcattctaagaccttttgttaaa

2447 ttattttaaagcatttcactagtatttcattctaagaccttttgttaaa

2602 tagtttcaagtttcaagttttacttttattaatgttttagaacatgtta

2496 tagtttcaagtttcaagttttacttttattaatgttttagaacatgtta

2651 atgtgtctaacggtcatacttgctctcacctcactcatctattgtgttt

2545 atatgtctaacggtcatacttgctctcacctcactcatctattgtgttt

2700 acatatatggctaaaatgacctatgcgtgtgtgaggagggccatgttga

2594 acatatatggctaaaatgacctatgcgtgtgtgaggagggccatgttga

2749 gagacttagtccctcataaatatttgttgttcacgtagaaagatgttat

2643 gagacttagtccctcataaatatttgttgttcacgtagaaagatgttat

2798 gtgaatgtaaactttgaattatgtatgcaggcttaaacaggtgcaggaa

2692 gtgaatgtaaactttgaattatgtatgcaggcttaaacaggtgcaggaa

2847 gagctgcagacaaagatggttaaactatctgaagccaaatatcaagaga

2741 gagctgcagacaaagatggttaaactatctgaagccaaatatcaagaga

2896 ggagactttaaagaggatgaagtagatcttataattagacttcacaggc

2790 ggagactttaaagaggatgaagtagatcttataattagacttcacaggc

2945 ttttgggaaacaggtactaataaataagtgtcattttcaattcatgtcg

2839 ttttgggaaacaggtactaataaataagtgtcattttcaattcatgtcg

2994 tcgttttcattgtacggaaattggacctattaacagtgagattataatc

2888 tcgttttcattgtacggaaattggacctattaacagtgagattataatc

3043 atagacctcaaactactttttccactcttttaatattttaatgtttttc

2937 atagacctcaaattactttttccactcttttaatattttaatgtttttc

3092 aatgaagtattagtggtgtgtagaataaaaaataaataaaaggtgtggt

2986 aatgaagtattagtggtgtgtagaatataaaaaaaataaaaggtgttgt

3141 gtaagtaatttgaagtatgtgaatataatatttcttgttaatatattct

3035 gtaagtaatttggagtgtgtgaatataatctttcttgttaatatattct

3190 ggctccccatattttcagtattttctaatacttcctaatttatatgtca

3084 ggctccccatattttcagtattttctaatacttcctaatttatatgtca

3239 ttttatttttcatttagacatcaagcaaaaagttttcaattttgtagta

3133 ttttatttttcatttagacatcaagcaaaaagttttcaattttgtagta

3288 tttttttagatttattaaaaacaattatttcccaaatttttttgtgggc

3182 tttttttagatttattaaaaacaattatttcccaaatttttttgtgggc

3337 caatggcctaccacatcattgtttaatggagaacttaaaggctagagta

3231 caatggcctaccacatcattgtttaatggagaacttaaaggctagagta

3386 acgaagtatgattttagagcaaatcgtaattttaggtataaaagtgaga

3280 acgaagtatgattttagagtaaatcgtaattttaggaataaaagtgaga

3435 ggagaaaaactaagggtagcaacgtgcaaatttcacgatacttgagtat

3329 ggagaaaaactaagggtagcaacttgcaaatttcatgatacttgagtat

3484 agtaaagtgaggattactcttattttttagctatagtctagcatgagaa

3378 agtaaagtgaggattactcttattttttagctatagtctagcatgagaa

3533 tctaaactacaaaatcattagagagggcaagcgttataaacattcattt

3427 tctaaactacaaaatcattagagagggcaagcgttataaacattcattt

3582 taaattttttaatattataatattctaccttaaggggcagagttgtttt

3476 taaattttttaatattataatattctaccttaaggggcagagttgtttt

3631 ttggttaagcaaaacaaaaaatcattcgtatcaaatgtggtatcaatga

3525 .tggttaagcaaaacaaaaaatcattcgtatcaaatgtggtatcattga

3680 aaatcaaacttcagactttagtcttaatttttaaaatgaagacaaatat

3573 aaatcaaacttcagactttagtcttaatttttaaaatgaagacaaatat

3729 cgagtgctaatagca....accaaaaattttaggaactgtttgatatct

3622 cgagtgctaatagcaataaaccaaaaattttaggaactgtttggtatct

3774 tatttgaaattttttatcatttctctaaacatttcttaaaaacatttct

3671 tatttgaaa.tttttatcatttctcaaaacattt.ttaaaaacatttct

3823 tgaaaacaattttctttaagacgcaaaaacttgatgagtatgcaaatta

3718 tgaaaacaattttctttaagactcaaaaacttgatgggtatgcaaatta

3872 aaaatttcaaatcttacgactttaactagacaaagagatctaaac.aga

3767 aaaatttcaaatcttacgactttaactagacaaagagatctaaacgaga

3920 gggggtcacggtagagggagaaagaag..ataggaggaaaaaagtaaga

3816 .ggggtcacggtagagggagaaagaagagagaggaggaaaaaagtaaga

3967 gatgattgaaagaaagaaaaataagagagatgatccgatgagatagaga

3864 gatgattgaatgaaagagaaagaagagagatgatcggatgaaatagaga

4016 ggaatatgagtgagagaaagaaccgagaggataaaaagagcagattgga

3913 ggaatatgagtgagagaaagaaccgagaggataaa.agagcagattgga

4065 gaaaagacgaaaaggta..aaaaaaaaggagagagaaagaagaaaagag

3961 gaaaagacgaaaaggtaataaaaaaaaggagagagaaagaagaaaagag

4112 agagatagatttggagagagagaggaggagggagagaaaagaaataaga

4010 agagatagatttggagagagagaggaggaagaagagaaaataaataaga

4161 gattttaagtttaaaaactctaaaactcacttttatgtttttagataat

4059 gagtttaagtttaaaaactctaaaactcacttttatgtttttagataat

4210 agactatattttt.agttagtcttgagttcaatttttaaaaatagtcct

4108 agactatatttttgagttagtcttgagttcaatttttaaaaatagtcct

4258 accaaacaagtttttaaggcctaaaacttgaaaattgtttttgagttta

4157 accaaa.aagtttttaaggcctaaaacttgaaaattgtttttgagttta

4307 aaaagttggattcaaataaagtaccaaacaagtccttagtttttcttga

4205 aaaagttggattcaaataaagtatcaaac.agtccttagtttttcttga

4356 ccgaaaaaataaaaatctttatccggaagggcattagtaaactcaaaca

4253 ccgaaaaaataaaaatctttatccggaagggcattagtaaactcaaaca

4405 acctttcttcgtaatgatttttgtatgtaaagtcattttcatgctttta

4302 acctttcttcgtaatgatttttgtatgtaaagtcattttcatgctttta

4454 atccctagtcgactgagcaaacctttaagattgtgtattcggcctacag

4351 atccctagtcgactgagcaaacctttaagattgtgtattcggccaacag

4503 aggcttggatcagaatagataagaagttatacattcaaaatttcacaat

4400 aggcttggatcagaatagataagaagttatacattcaaaatttcacaat

4552 taataaaattagaagggagaactttggtaataaatacacgcagtaattt

4449 taatagaattagaagggagaactttggtaataaatacacgcagtaattt

4601 tatttttgtattaaaactaatgttcgggcaaggatttggcctttgcaca

4498 tatttttgtattaaaactaatgttcgggcaaggatttggccttggcaca

4650 gctcccttggagtgttggcacttggtgttgatgttggttgttggtcgag

4547 gctcccttggagtgttggcacttggcgttgatgttggttgttggtcgag

4699 ttcttgctacatggtgtgctacaagaagagtacaaagttagttttg.at

4596 ttcttgctacttggtgtgctacaagaagagtacaaagttagttttgaaa

4747 tgtgcctttgtggggctttagatgtaggtcttgaggctcacaatcaaaa

4645 tgtgcctttgtggggccttagatgtaggtcttgaggctcacaatcaaaa

4796 ctaacaaagagttaggcgtgccactgttatctcaatataatagatgttg

4694 ctaacaaaaagttaggcgtgccactgttatctcaatataatagatgttg

4845 aatatatttgatctaagtcgattacttgcgccctaagatgcttaaactt

4743 aatatatttgatctaagtcgattacttgcgccccaagatgcttaaactt

4894 cttgttatcaaaggattgtcacaaatgggttaagtctgtattaagttct

4792 cttattatcaaaggattgtcacaaatgggttaagtc.gcattaagttct

4943 ttttcttgccttgtgaccaaggactttttcttatagttttgtatgttta

4840 ttttcttgccttgtgaccaaggactttttcttatagttttgtatgttta

4992 gatgaagggagtccagaacccttttaatcattcgcttgattacaattcg

4889 gatgaagggagtccataacccttttaatcattcgcttgattacaattcg

5041 acgcttaaagaagtgaagttaacttgcttagccaaatttgatcataaat

4938 acgcttaaagaagtgaagttaacttgcttagccaaatttgatcataaat

5090 gggtcttaaagcgagaaacaattgcaagtgagttaaaacataacagaat

4987 gggtcttagagcgagaaacaattgtaagtgagttaaaacataacataat

5139 atgcatttaaacaacagatctacaaactgtaagtacaaacacaaggaag

5036 atgcatttaaacaacaaatctacaaac.gtaagtacaaacacaaggaag

5188 ttgggcaagatttcaccttgtcgggcaaagttcaaacctcttgcaacca

5084 ttgggcaagatttcatcttgtcgggcaaggttcaaacctcttgcaacca

5237 cttctctttgagtttgtagaagagttgtgggta.ttgcaaaatggaaat

5133 cttctccttgagtttgtagaagagttgtgggtacttgcaaaatggaaat

5285 gtaagcaagaaatacaaataaggtttcctaaagggaatctattctacga

5182 gtaagcaggaaatacaaacaaggtttcctaaagggaatctattctacga

5334 atctaggataaggtagcagagctttgccaaaagatgactttctgcgtgg

5231 atctaggataaggtagc.gagctttgccaaaagatggctttc.gcgtgg

5383 aatctatggctaaaaggtgcagaatctggacaaagtgcagctttttggg

5278 aatctatggctaaaaggtgcataatc.ggacgaagtgcagctttttggt

5432 tatttgcttgtctgagaggcaagtttgtatgtcttttgtttgattggtt

5326 tatttgcttgtc.gagaggcaagtttgtatgtcttttgtttgattggtt

5481 gagtgtccttgtctctgttgtctctttctccttttatagacgatttggc

5374 gagtgtccttgtctctgttgtctctttctccttttatagacgatttggc

5530 ccgactgcttttagctctattcttgtccgaaagctcttggagggcaatg

5423 ccgac.gcttttggctctattcttgtccgaaagctcttggagggcaatg

5579 agtcatcatctttttacttgtagtgccattagaaagtgttttttggcta

5471 agtcatcatctttttacttgtagtgccattagaaagtgttttttggcta

5628 atagtgagttggtctctgtcacttgtcacttcactcctacacatgtgtg

5520 atagtgagttgatctctgtcacttgtcacttcactcctacacatgt..g

5677 gcctatattttaattgaggaggcaccaatttgttacaggcttgtcgact

5567 gcctatattttaattgaggaggcaccaatttgttccaggcttgtcgact

5726 gggcctcgggcaagtcttcacttaatttgacatccatgggccttgacta

5616 gggcctcgggcaagtcttcacttaatttgacatccatgggtcttggcta

5775 tttacaaaaccctatgttaaatattaactcaaacaactagtccactcca

5665 tttacaaaaccttatgttaaatattaactcaaacaactagtccactcca

5824 tttaattctaaagaagaaaatcgtttatgcaatctctgtt.cttttttt

5714 tttaattctaaagaagaaaatcgtttatgcaatctctgttccttttttt

5872 tctttattcatcttatttttcaggcaaatgtattcatcatttttcttca

5763 tctttattcatcttatttttcaggcaaatgtattcatcatttttcttca

5921 tgcatgtaatgaacttaggtggtcattgattgctagaagacttccagga

5812 tgcatgtaatgtacttaggtggtcattgattgctagaagacttccagga

5970 agaacagcaaatgctgtgaaaaattattggaacactcgattgcggatcg

5861 agaacagcaaatgctgtgaaaaattattggaacactcgattgcggatcg

6019 attctcgcatgaaaacggtgaaaaataaatctcaagaaatgagagagac

5910 attctcgcatgaaaacggtgaaaaataaatctcaagaaatgagaaagac

6068 caatgtgataagacctcagccccaaaattcaacagaagttcatattact

5959 caatgtgataagacctcagccccaaaattcaacagaagttcatattact

6117 taagcagtaaagaaccaattctagaccatattcaatcagcagaagattt

6008 taagcagtaaagaaccaattctagaccatattcaatcagcagaagattt

6166 aagtacgccaccacaaacgtcgtcgtcaacaaagaatggaaatgattgg

6057 aagtacgccaccacaaacgtcgtcgtcaacaaagaatggaaatgattgg

6215 tgggagaccttgttagaaggtgaggatacttttgaaagagctgcatatc

6106 tgggagaccttgttagaaggcgaggatacttttgaaagagctgcatatc

6264 ccagcattgagttagaggaagaactcttcacaagtttttggtttgatga

6155 ccagcattgagttagaggaagaactcttcacaagtttttggtttgatga

6313 tcgactgtcgccaagatcatgcgccaattttcctgaaggacaaagtaga

6204 tcgactgtcgccaagatcatgcgccaattttcctgaaggacaaagtaga

6362 agtgaattctcctttagcacggacctttggaatcattcaaaagaagaat

6253 agtgaattctcctttagcacggacctttggaatcattcaaaagaagaat

6411 agctagagaaaatgattctcacttctgtagtatcatctagcttgtgtac

6302 agctagagaaaatgattctcacttctttattatcatctagcttgtgttc

6460 tattattttccttgcttgtaaatgtggcatgtaaatatcattaagcttg

6351 tattattttccttgcttgtaaatgtggcatgtaaatatcattaagcttg

6509 atgaaattgagattccaccataaaaccaattggaaatatggagagtagc

6400 atgaaattgagattccaccataaaaccaattggaaatatggggagtagc

6558 ccaagaccatataagcacatagcaaaccttgtccctcaccgatgtggta

6449 ccaagaccatataagcacatagcaaaccttgtccctcaccgatgtggta

6607 caactgtcaacacaccctcgcatgtgtggcagattttcaagcctacacg

6498 caactgtcaacacgccctcgcatgtgtggcagattttcaagcctacaca

6656 tggataacaaccgggtgacgtggagcgcatgtggccatttggcttcaca

6547 tggacaacaa.cgggtgacgtggagcgcatgcggccatttggcttcaca

6705 cgaggacaacccgctctaataccatgatgaaattaaggatccaccgtaa

6595 cgaggacaacccgctc.gataccatgatgaaattaaggttccaccataa

6754 aactaattggtaatatgaggagtagcctaagaccatataagcacatagc

6643 aaccaattggtaatatggggagtaggctaagaccatataagcacatagc

6803 aaaccttgttcctcaccgatatgggacaactgtcaacaaagc.gacgag

6692 aaaccttgtccctcaccgatatggaacaactgtcaacaaagctgacgag

6851 gaatcctaatcactggacttattttaataagtgaactatttttaatagt

6741 gaatcctaatcactggacttattttaataagtgaactatttttaatagt

6900 ggttcacaaaattggacttaaatcaaattttctcaaaataaattcagca

6790 ggttcacaaaattggacttaaatcaaattttctcaaaataaattcagca

6949 actaccacaaccaagttctttcatgtacaaatgtggctgaatcatataa

6839 actaccacaaccaagttctttcatgtacaaatgtggctgaatcatataa

6998 gcaataaaaactaacaacttaaaggaaattctatacttgtaatcttctg

6888 gcaataaaaactaacaacttaaaggaaattctatacttgtaatcttctg

7047 cattttcaaagattccaaaggtcctccataaaggagaagcccctgccac

6937 cattttcaaagattccaaaggtcctccataaaggagaagcccctgccac

7096 tccttaccttcatcaaaactgccacatgatcttgttaattggaccctag

6986 tccttaccttcatcaaaactgccacatgatcttgttaattggaccctag

7145 acttcaacccaaaagtctgaatggatctttcatgtaatccaagactaga

7035 acttcaacccaaaagtctgaatggatctctcatgtaatccaagactaga

7194 aattgttgttctgtgaagtacactatcggggatctcatttccaacaacc

7084 aattgttgttctgtgaagtacactatcggggatctcatttccaacaacc

7243 agtttgattctctattggatgcgatcatccttgcattttttactacaat

7133 agtttgattctctattggatgcgatcatccttgcaatttttgctacaat

7292 ggttttctgattaattttgttctgcaatcaccacttctcaaacgcagtg

7182 ggttttctgattaattttgttctacaatcaccacttctcaaacgcagtg

7341 aactatatattgaagaaggtttgtctaaagaaaaatggaagtgtttcca

7231 aactatatattgaagaaggtttgtctaaagaaaaatggaagtgtttcca

7390 cgagaaagtgtagacaaaatcatgtctatgaacaacagttccaattaaa

7280 cgagaaagtgtagacaaaatcaagtctatgaacaacagatccaattgaa

7439 gaaaatcatctagtctcagaggttgcggcgttacaatgctccaaatgca

7329 gaaaatcatctactctcagaggttgtggcgttacaatgctccaaatgca

7488 tacgataatgcaaaatcatgggattaatttcacagaaattgtagacaaa

7378 tacgataatgcaaaatcatgggattaatttcacaaaaattgtagacaaa

7537 atcacataatctacaaacccctagagccaggaacatcaatccactgtaa

7427 atcacataatctacaaacccctagagccaggaacatcaatccactgtaa

7586 ttggagctccagttccccactctccacatttctcagcctgatacacata

7476 ttggagatccagttccccactctccacatttctcagcctgatacacata

7635 ttctggacaacttggccatcagtatggacgatgcagctctcttcagcta

7525 ttctggacaacttggccatcagtatagacgatgcagctctcttcagcta

7684 gacagttttgccggcttggttggaatcttgaaattatggttccacttgg

7574 gacagttttgccggcttggttggaatcttgaaattatggttccatttgg

7733 tacgccttgcaaacccacacgtaaagcttgaacaaaaggaccaatctca

7623 tacgccttgcaaacccacacgtagagcttgaacaaaaggaccaatctca

7782 aactcagcatcccccatcttgtcatcaagactaaacgtgtctttgtcat

7672 aactcagcatcccccatcttgtcatcaagactaaacgtgtctttgtcat

7831 acacaaactgcaatataagcaccccaaaaaattcagaacaatgtccaaa

7721 acacaaactgcaatataagcaccccaaaa.attcagaacaatgtccgaa

7880 gtttcatttcatgcctcacacaggccgaaaaatctcaggaccagccctg

7769 gtttcatttcatgcctcacacaggccgaaaaatctcaggaccagccctg

7929 tatgcgaaagacaatcaatccagaatgtttgatagaaaatagtacttac

7818 tatgcgaaagacaatcaatccagaatgtttgatagaaaatagtacttac

7978 aagtttgataggaaggcttggatctgcaaccgaaagagttaagtcttcg

7867 aagtttgataggaaggcttggatctgcaaccgaaagagttaagtcttcg

+7873 ttccac^

**PAM**

-- END alignment [ +1 2112 - 8026 | +1 2006 - 7915 ]
